# Additivity and Chain Rules for Quantum Entropies via Multi-index Schatten Norms

**DOI:** 10.1007/s00220-026-05567-8

**Published:** 2026-03-09

**Authors:** Omar Fawzi, Jan Kochanowski, Cambyse Rouzé, Thomas Van Himbeeck

**Affiliations:** 1 ENS Lyon, UCBL, LIP, Inria, 69342 Lyon Cedex 07, France; 2 Télécom Paris - LTCI, Institut Polytechnique de Paris, Inria, 91120 Palaiseau, France; 3 Inria Paris, Paris, France

## Abstract

The primary entropic measures for quantum states are additive under the tensor product. In the analysis of quantum information processing tasks, the minimum entropy of a set of states, e.g., the minimum output entropy of a channel, often plays a crucial role. A fundamental question in quantum information and cryptography is whether the minimum output entropy remains additive under the tensor product of channels. Here, we establish a general additivity statement for the optimized sandwiched Rényi entropy of quantum channels. For that, we generalize the results of Devetak et al. (Commun Math Phys 266(1):37–63, 2006) to multi-index Schatten norms. As an application, we strengthen the additivity statement of Van Himbeeck and Brown (A tight and general finite-size security proof for quantum key distribution, 2025) thus allowing the analysis of time-adaptive quantum cryptographic protocols. In addition, we establish chain rules for Rényi conditional entropies that are similar to the ones used for the generalized entropy accumulation theorem of Metger et al. (Commun Math Phys 405(11):261, 2024).

## Introduction

Entropy is a cornerstone of information theory, governing fundamental limits in communication, compression and statistical inference. Entropic quantities often behave extensively when evaluated on composite systems, a property encapsulated by chain rules, additivity or uncertainty relations. Yet, establishing such statements often presents significant challenges, a fact that is particularly true in the quantum setting. A powerful perspective emerges by recognizing that entropies naturally arise as logarithms of certain $L_p$-norm quantities, allowing deep functional analytic methods to be used in information theory, for example to establish *entropy power* inequalities or *uncertainty principles*–closely tied to the behavior of $L_p$ norms under convolution or Fourier transform [9, 16].

Of particular importance to cryptography is the conditional $\alpha$-Rényi entropy of a bipartite distribution $p_{AB}$, $\alpha \ge 1$ [1]:

$$\begin{aligned} H^{\uparrow }_\alpha (A|B)_p:=\frac{\alpha }{1-\alpha }\log \left[ \sum _b p(b)\, \left( \sum _ap(a|b)^\alpha \right) ^{\frac{1}{\alpha }}\right] \,. \end{aligned}$$

In the above, the quantity inside the logarithm can be interpreted as the $\ell _{(1,\alpha )}$-norm of the function $(a,b)\mapsto p(a,b)$. Similarly, multipartite extensions of conditional Rényi entropies would involve multi-index $\ell _p$ norms: given a vector $v\in \bigotimes _{i=1}^k\mathbb {C}^{d_i}$ and indices $\{p_i\}_{i=1}^n$ the $\ell _{(p_1,...,p_k)}$ norm of *v* is defined as

$$\begin{aligned} \Vert v\Vert _{(p_1,...,p_k)} := \left( \sum _{a_1=1}^{d_1}\left( \sum _{a_2=1}^{d_2}\,... \left( \sum _{a_k=1}^{d_k}\big |v_{a_1a_2...a_k}\big |^{p_k}\right) ^{p_{k-1}/p_k} ...\,\right) ^{p_1/p_2}\right) ^{{1/p_1}}\,. \end{aligned}$$

While this correspondence is straightforward in the classical setting, it becomes more subtle in the quantum case due to the absence of a preferred basis. Note that in the case of a single index, i.e., $k=1$, the Schatten norm ${{\,\mathrm{\mathcal {S}}\,}}_p(\mathbb {C}^d)$ defined by $\Vert X \Vert _{p} = \hbox {tr}[|X|^p]^{1/p}$ is a natural non-commutative extension of the norm $\ell _p$. However, the multi-index, i.e., $k \ge 2$ case, is more difficult as direct extensions such as

$$\begin{aligned} (\hbox {tr}_1([\hbox {tr}_2[...(\hbox {tr}_k[|X|^{p_k}])^{{p_{k-1}/p_k}}...])^{{p_1/p_2}}])^{{1/p_1}} \end{aligned}$$

for operators $X$ acting on $\bigotimes _{i}\mathbb {C}^{d_i}$, do not define norms [10]. As an attempt to restore the norm property via operator space theoretic methods, Pisier introduced the concept of *operator-valued Schatten norms* in his seminal work [20]. An operator space ${{\,\mathrm{\mathcal {X}}\,}}$ consists of a family of norms $M_{n}({{\,\mathrm{\mathcal {X}}\,}})$ indexed by *n* on the space of $n \times n$ matrices with entries in ${{\,\mathrm{\mathcal {X}}\,}}$. Such a family should satisfy natural conditions for an operator norm. For an arbitrary operator space ${{\,\mathrm{\mathcal {X}}\,}}$, Pisier proposed the following extension of *p*-Schatten norms to $M_{d_1}\otimes \mathcal {X}$, whose form is reminiscent of Hölder’s inequality:

$$\begin{aligned} \Vert X\Vert _{\mathcal {S}_{p_1}[\mathbb {C}^{d_1},\mathcal {X}]}= \inf _{\underset{X=FYG}{F,G\in \mathcal {S}_{2p_1}(\mathbb {C}^{d_1}), Y\in M_{d_1}({{\,\mathrm{\mathcal {X}}\,}})}}\Vert F\Vert _{2p_1}\Vert Y\Vert _{M_{d_1}({{\,\mathrm{\mathcal {X}}\,}})}\Vert G\Vert _{2p_1}\,, \end{aligned}$$

Choosing $\mathcal {X}$ itself as a Schatten space $\mathcal {S}_{p_2}(\mathbb {C}^{d_2})$, Pisier’s formula provides a means to define a version of $\ell _{(p_1,p_2)}$ for operators (see Theorem 2.5 below). By iterating this procedure, one gets a natural quantum extension of $\ell _{(p_1,\dots \,p_k)}$:

$$\begin{aligned} \Vert X\Vert _{(p_1,p_2\cdots \, p_n)} = \Vert X\Vert _{\mathcal {S}_{p_1}[ \mathbb {C}^{d_1}, \mathcal {S}_{p_2}[ \mathbb {C}^{d_2} \cdots \, \mathcal {S}_{p_n}(\mathbb {C}^{d_n})]\cdots ]}\,. \end{aligned}$$

A major connection between these multi-index Schatten norms and quantum entropies was established in [10]: the $(1,\alpha )$-Schatten norm can be related to von Neumann entropies by taking appropriate derivatives. Later, the sandwiched Rényi conditional entropies were defined as

$$\begin{aligned} H^\uparrow _\alpha (A|B)_\rho := -\inf _{\sigma _B\in \mathcal {D}(\mathcal {H}_B)}D_{\alpha }(\rho _{AB}\Vert \mathbb {1}_A\otimes \sigma _B), \end{aligned}$$

where $D_\alpha$ corresponds to the sandwiched Rényi divergence of order $\alpha \ge 1$ [19, 24, 27] (see Sect. 2.5). As observed in [6], such conditional entropy can directly be related to $(1,\alpha )$-Schatten norms:

1 $$\begin{aligned} H^\uparrow _\alpha (A|B)_\rho = \frac{\alpha }{1-\alpha }\log \Vert \rho _{BA}\Vert _{(1,\alpha )}. \end{aligned}$$

### Main results

#### Multiplicativity of completely bounded norms

In addition to pointing out the deep connection between Pisier spaces and conditional quantum entropies, in [10], the authors proved the multiplicativity of the completely bounded norms between $L_p$ spaces for completely positive (CP) maps: in particular, given two channels $\Phi _1:Q_1\rightarrow S_1$, $\Phi _2:Q_2\rightarrow S_2$ and any $1\le p,q \le \infty$, [10] showed that the product channel $\Phi =\Phi _1\otimes \Phi _2:Q\rightarrow S$, with $Q = Q_1Q_2$ and $S=S_1S_2$ satisfies

$$\begin{aligned} \Vert \Phi \Vert _{cb, \mathcal {S}_p(\mathcal {H}_{Q}) \rightarrow \mathcal {S}_q(\mathcal {H}_{S})} = \Vert \Phi _1\Vert _{cb, \mathcal {S}_p(\mathcal {H}_{Q_1}) \rightarrow \mathcal {S}_q(\mathcal {H}_{S_1})}\Vert \Phi _2\Vert _{cb, \mathcal {S}_p(\mathcal {H}_{Q_2}) \rightarrow \mathcal {S}_q(\mathcal {H}_{S_2})}\,, \end{aligned}$$

where $\Vert \cdot \Vert _{cb,\mathcal {X}\rightarrow \mathcal {Y}}$ denotes the completely bounded norm between two operator spaces. Generalizing the multiplicativity to arbitrary multi-index Schatten operator spaces, we prove in Theorem 4.7, for any CP maps $\{\Phi _i:Q_i\rightarrow S_i\}$ and numbers $1\le q_i,p_i\le \infty$,

$$\begin{aligned} \bigg \Vert \bigotimes _{i=1}^n\Phi _i\bigg \Vert _{cb,(q_1,...,q_n)\rightarrow (p_1,...,p_n)} = \prod _{i=1}^n\Vert \Phi _i\Vert _{cb,q_i\rightarrow p_i}\,. \end{aligned}$$

#### Additivity of output $\alpha$-Rényi conditional entropy

Reinterpreting the multiplicativity result of [10] in terms of Rényi $\alpha$-entropies and choosing $(p,q) = (1,\alpha )$ yields the additivity of the output Rényi $\alpha$-entropy of CP maps:

$$\begin{aligned} \inf _E\inf _{\rho _{EQ}}H_\alpha ^\uparrow (S|E)_{\Phi (\rho )}=\inf _E\inf _{\rho _{EQ_1}}H_\alpha ^\uparrow (S_1|E_1)_{\Phi _1(\rho )}+\inf _E\inf _{\rho _{EQ_2}}H_\alpha ^\uparrow (S_2|E_2)_{\Phi _2(\rho )}\,. \end{aligned}$$

In [25] using different techniques, the authors proved that for a CP map $\Phi :Q\rightarrow RS$ with classical output registers *R*, *S*, the *n*-fold tensor product map $\Phi ^{\otimes n}: Q^n\rightarrow R^nS^n$ satisfies

$$\begin{aligned} \inf _E\inf _{\rho _{EQ^n}}H_\alpha ^\uparrow (S^n|R^nE)_{\Phi ^{\otimes n}(\rho )}&= n \cdot \inf _E\inf _{\rho _{EQ}}H_\alpha ^\uparrow (S|RE)_{\Phi (\rho )}\,. \end{aligned}$$

This result was referred to as IID reduction since it implies that the minimizer of the LHS takes the tensor product form $\rho _{E^\prime Q}^{\otimes n}$ with $E = {E^\prime }^n$, representing identically and independently distributed quantum systems. In terms of operator norms, note that this is equivalent to $1\rightarrow (1,p)$ norms $\Vert \Phi ^{\otimes n}\Vert _{cb, 1 \rightarrow (1,p)} = \Vert \Phi \Vert _{cb, 1 \rightarrow (1,p)}^n$.

We generalize both results and show in Theorem 4.11 that for any CP maps $\Phi _i:Q_i\rightarrow R_iS_i$, the product channel $\Phi ^n=\bigotimes _{i\le n} \Phi _i:Q^n\rightarrow R^n S^n$ with $Q^n = Q_1\cdots Q_n$, $R^n = R_1\cdots R_n$, $S^n=S_1 \cdots S_n$, satisfies

2 $$\begin{aligned} \inf _E\inf _{\rho _{EQ^n}}H_\alpha ^\uparrow (S^n|R^nE)_{\Phi ^n(\rho )}&= \sum _i \inf _E\inf _{\rho _{EQ_i}}H_\alpha ^\uparrow (S_i|R_iE)_{\Phi _i(\rho )}\,. \end{aligned}$$

This is equivalent to $\Vert \Phi ^n\Vert _{cb, 1 \rightarrow (1,p)} = \prod _{i=1}^n \Vert \Phi _i\Vert _{cb, 1 \rightarrow (1,p)}$. Note here the difference between the product channel $\Phi ^{\otimes n}$ made up of *n* copies of the same map, considered in [25] and the more general product of *n* different channels denotes as $\Phi ^n$. We will continue this notation convention throughout this work.

#### Chain rule for the $\alpha$-Rényi entropy

As a consequence of the chain rule for $\alpha$-Rényi entropies derived in [17, Lemma 3.6], for any two channels $\Phi _1:Q_1\rightarrow S_1$ and $\Phi _2:Q_2\rightarrow S_2$ with $\Phi =\Phi _1\otimes \Phi _2$, $\alpha \in (1,2)$ and any state $\rho _{QT}$,

$$\begin{aligned} H_\alpha (TS_2|S_1)_{\Phi (\rho )}\ge H_\alpha (T|Q_1)_\rho +\inf _{\sigma _{Q\tilde{Q}}}H_{\frac{1}{2-\alpha }}(S_2|S_1\tilde{Q})_{\Phi (\sigma )} \end{aligned}$$

for a purifying system $\tilde{Q}$ of $Q=Q_1Q_2$, where we recall that the non-optimized Rényi conditional entropy is defined as $H_\alpha (A|B)_\rho :=-D_\alpha (\rho _{AB}\Vert \mathbb {1}_A\otimes \rho _B)$, and where the infimum on the right-hand side is over all quantum states on $Q\tilde{Q}$.^1^ Exploiting operator valued Schatten spaces, we derive a similar, yet seemingly tighter inequality for the optimized Rényi entropy (see Corollary 4.9): for any $\alpha \ge 1$ and any state $\rho _{QT}$,

3 $$\begin{aligned} H^\uparrow _\alpha (TS_2|S_1)_{\Phi (\rho )}\ge H_\alpha ^\uparrow (T|Q_1)_\rho +\inf _{\sigma _{Q_1Q_2\tilde{Q}}}H^\uparrow _\alpha (S_2|S_1\tilde{Q})_{\Phi (\sigma )}. \end{aligned}$$

Moreover, we derive the following variant for two-output channels (see Corollary 4.3): For any state $\rho _{QT}$ and any quantum channel $\Phi :Q\rightarrow RS$,

4 $$\begin{aligned} H^{\uparrow }_\alpha (TS|R)_{\Phi (\rho )} - H^{\uparrow }_\alpha (T|Q)_\rho&\ge \inf _{\sigma _Q} H^{\uparrow }_\alpha (S|R)_{\Phi (\sigma )}, \end{aligned}$$

Once again, this bound can be compared to [17, Lemma 3.6]: while our result is less general, it is directly stated for the optimized Rényi entropy, suffers no loss in the parameter $\alpha$, and does not require optimizing over purifications on its right-hand side.

#### Application to quantum cryptography

Quantum entropies have many applications in quantum cryptography and quantum key distribution [29] in particular. Historically, their theoretical study has gone hand in hand with the development of rigourous and efficient security proof for QKD [2, 18, 23, 25]. In the present article, we follow this trend and show (Theorem 5.2) how our new additivity results for output Rényi entropies lead to a new family of security proofs for quantum protocols that are time-dependent and that are subjected to time-dependent experimental conditions.

In quantum key distribution, the assumption is usually made that the protocol does not vary with time. This setting is well-adapted to static scenarios, such as protocols deployed over optical fibers where fluctuations can be neglected. This is not the case however for free-space implementations such as satellite QKD [15] where the trajectory of the satellite and atmospheric turbulence lead to noise patterns that vary with time.

It is possible to apply a static security proof in cases where the noise on the channel varies but not the protocol itself. While the security statements will not be affected in this case, the performance may not be optimal. Here, we show that in cases where the noise varies with time in a way that is predictable, we can achieve higher secret key rates than with traditional static security proofs. Moreover, our new security proof also applies to protocols that change over times, which opens up the possibility of designing new QKD protocols that are specially tailored to time-dependent scenarios.

### Structure of the paper

Each of our main results is stated in two ways, highlighting the correspondence between the functional and entropic settings discussed above: first in terms of submultiplicativity of (completely bounded) operator norms between operator-valued Schatten spaces of CP maps, then via (1) as inequalities for the output Rényi conditional entropy of quantum channels.

In Sect. 3, we recall the main notions of Pisier’s formalism, and in particular Theorem 2.3, which we extensively use to derive general variational formulas with the goal to make triple-index Schatten norms more tractable. We do this by introducing a systematic way to derive variational formulas for Schatten norms for arbitrary numbers of indices in Lemma 3.1, and later focus on the case of 3 indices in Theorem 3.2 and Theorem 3.4.

In Sect. 4, we apply these bounds to derive our main results. First, we derive in Theorem 4.1 a generalization of [10, Lemma 5] to two-output CP maps of the form $\Phi :Q\rightarrow RS$. Informally, this result states that “identities to the right” do not have an effect on CP maps between operator-valued Schatten spaces. The chain rule that results from this is stated in Corollary 4.5, see also (4). We then prove a general ordered multiplicativity result for completely bounded norms in Theorem 4.7, which follows from the aforementioned Theorem 4.1. A direct consequence of it is the entropic chain for product maps already stated in (3), see Corollary 4.9. Our last main technical result is a (non-ordered) multiplicativity result for $1\rightarrow (1,p)$-completely bounded norms in Theorem 4.11. We further show multiplicativity under arbitrary linear input constraints Theorem 4.16 and for weights to get the additivity statement for the minimum output entropy Corollary 5.1. This is the generalization to tensor products of arbitrary quantum channels of the IID reduction from [25] already hinted at in (2). This result is applied to quantum key distribution in Sect. 5.

**Fig. 1:**
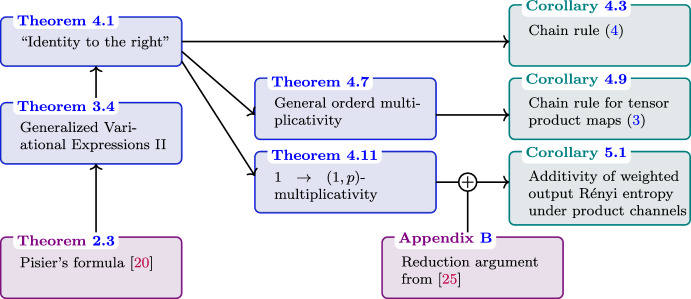
The above figure illustrates the main implications presented in this work, excluding the applications to QKD. Violet boxes represent external results, blue boxes our main theorems presented in terms of channel norms, and teal boxes their transcription in terms of conditional Rényi-entropies

For an overview of these results and their connection, see Fig. 1.

We note that Arqand and Tan [3] independently obtained similar results using different techniques.

## Preliminaries

The aim of this section is to give preliminaries and set notations for this article. For Sect. 3 in particular we require notions of operator spaces and operator-valued Schatten norms. The required notions will be introduced in Sects. 2.2 and 2.3. There in particular we also introduce new notations for multi-index Schatten norms, which we believe to be well suited in the context of quantum information theory.

The proofs of most of the statements can be found in the main body of the text, however, proofs for either well-known facts, or ones that are very similar to proofs in the main body are presented in the appendix.

### Basic notation

We denote by $[n]:=\{1,...,n\}$ the set of natural numbers until $n\in \mathbb {N}$. Quantum systems are denoted by upper case latin letters *Q*, *R*, *S*, while Hilbert spaces are denoted by $\mathcal {H}$, $\mathcal {K}$, $\mathcal {H}_R$, $\mathcal {H}_1$, etc., with norm denoted e.g. by $\Vert \cdot \Vert _{\mathcal {H}}$. They are assumed to be separable, unless explicitly stated to be finite dimensional. Given two Hilbert spaces, we denote with $\mathcal {H}\otimes \mathcal {K}$ the Hilbert space constructed as the completion of the algebraic tensor product of these two spaces with respect to the canonical norm induced by the tensor-product inner product on $\mathcal {H}\otimes \mathcal {K}$.

We denote the Banach space of bounded operators from some Hilbert space $\mathcal {H}$ to some other $\mathcal {K}$, i.e. $X:\mathcal {H}\rightarrow \mathcal {K}$, as $\mathcal {B}(\mathcal {H},\mathcal {K})$, with the operator norm $\Vert \cdot \Vert _\infty$. For simplicity we write $\mathcal {B}(\mathcal {H})\equiv \mathcal {B}(\mathcal {H},\mathcal {H})$. The identity element in $\mathcal {B}(\mathcal {H})$ is denoted by $\mathbb {1}\equiv \mathbb {1}_\mathcal {H}$. More generally, we often label an operator *X* supported on a labeled Hilbert space $\mathcal {H}_S$ as $X_S$. By slight abuse of notations, we will also denote by $X_S$ operators $X_S\otimes \mathbb {1}_R\in \mathcal {B}(\mathcal {H}_S\otimes \mathcal {H}_R)$ when clear from context. When $\mathcal {H}_S$ is of finite dimension, we sometimes denote its dimension by |*S*|.

An operator $X\in \mathcal {B}(\mathcal {H})$ is positive semidefinite, written $X\ge 0$, if it can be written as $X=Y^*Y$ for some other operator $Y\in \mathcal {B}(\mathcal {H})$, where $Y^*$ denotes the adjoint of *Y*. The set of all positive semidefinite operators acting on some Hilbert space $\mathcal {H}$ is denoted by ${{\,\textrm{Pos}\,}}(\mathcal {H})$. We denote $X>0$ if $X\ge 0$ and its kernel is trivial.

The Schatten-*p* space over $\mathcal {H}$ with index $1\le p\le \infty$ is denoted by $\mathcal {S}_p(\mathcal {H})$ with associated Schatten-norm $\Vert X\Vert _p:=\hbox {Tr}[|X|^p]^{\frac{1}{p}}$ when $p<\infty$ and $\Vert \cdot \Vert _\infty$ being the above mentioned operator norm, when $p=\infty$. $\mathcal {S}_p(\mathcal {K},\mathcal {H})$ is defined analogously. In both cases $\hbox {Tr}[\cdot ]$ is the canonical trace on $\mathcal {B}(\mathcal {H})$ and $|X|:=\sqrt{X^*X}.$ Note that $\mathcal {S}_\infty (\mathcal {H})$ coincides with the set of all compact operators endowed with the operator norm and that in finite dimensions we have $\mathcal {S}_\infty (\mathcal {H})=\mathcal {B}(\mathcal {H})$.

We will be denoting the partial trace as $\hbox {tr}_Q[\cdot ]:\mathcal {S}_1(\mathcal {H}_{QR})\rightarrow \mathcal {S}_1(\mathcal {H}_{R})$.

A trace-normalized, positive semidefinite, Schatten-1 operator is called a *quantum state*. We will usually denote such operator with lower case greek letters $\rho ,\sigma ,\omega$. We denote the set of all quantum states over some Hilbert space $\mathcal {H}$ as $\mathcal {D}(\mathcal {H}):=\{\rho \in \mathcal {S}_1(\mathcal {H} )|\hbox {Tr}[\rho ]=1, \rho \ge 0\}$.

A linear map $\Phi :\mathcal {B}(\mathcal {H})\rightarrow \mathcal {B}(\mathcal {K})$ is *completely positive (CP)* if ${{\,\textrm{id}\,}}_{\mathcal {B}(\mathbb {C}^n)}\otimes \Phi \in \mathcal {B}(\mathbb {C}^n\otimes \mathcal {H},\mathbb {C}^n\otimes \mathcal {K})$ is a positive map for all $n\in \mathbb {N}$. It is *trace preserving (TP)* if $\hbox {Tr}[\Phi (X)]=\hbox {Tr}[X]$ $\forall X\in \mathcal {S}_1(\mathcal {H})$. A *quantum channel* is defined as the restriction of a linear CPTP map to some state space $\mathcal {D}(\mathcal {H})$, i.e. it is an affine CPTP map $\Phi :\mathcal {D}(\mathcal {H}_Q)\rightarrow \mathcal {D}(\mathcal {H}_R)$. Its adjoint, denoted by $\Phi ^*:\mathcal {B}(\mathcal {H}_R)\rightarrow \mathcal {B}(\mathcal {H}_Q)$ is a linear, CP, unital (U) map, i.e. $\Phi ^*(\mathbb {1}_R)=\mathbb {1}_Q$. We denote the identity map as ${{\,\textrm{id}\,}}_S:\mathcal {B}(\mathcal {H}_S)\rightarrow \mathcal {B}(\mathcal {H}_S)$. To simplify notations, we also write $\Phi :Q\rightarrow R$ as a shorthand for the map $\Phi :\mathcal {B}(\mathcal {H}_Q)\rightarrow \mathcal {B}(\mathcal {H}_R)$.

### Operator spaces

In the following, we give a concise introduction into operator space theory and operator-valued Schatten norms. These will be a central tool to derive our chain rules and additivity results in Sects. 3 and  4. For a more complete review of operator space theory or operator valued Schatten spaces, see [6] or the books [20, 21].

Operator spaces originate from the study of non-commutative geometry. They are essentially concerned with the problem of providing natural norms on spaces of vector-valued matrices and the study of the resulting structures. Since we are studying composite quantum systems, we are concerned with matrix or operator-valued matrices, which is a prime application of operator space theory.

In the following, we let $\mathcal {X}\subset \mathcal {B}(\mathcal {K})$ be a linear subspace. Then we construct a “natural” family of norms on the spaces

$$\begin{aligned} M_{m,n}(\mathcal {X}):=\Big \{[X_{ij}]_{i\in [m],j\in [n]}|X_{ij}\in \mathcal {X}\Big \} \end{aligned}$$

of $\mathcal {X}$-valued $m\times n$ matrices. For simplicity we write $M_{n}(\mathcal {X})\equiv M_{n,n}(\mathcal {X})$. We construct norms on these spaces by viewing elements of $M_{m,n}(\mathcal {X})$ as linear maps in $\mathcal {B}(\mathcal {K}^n,\mathcal {K}^m)$, where $\mathcal {K}^n:=\bigoplus _{i=1}^n\mathcal {K}=\mathbb {C}^n\otimes \mathcal {K}$, via the identification $M_{m,n}(\mathcal {X})\subset M_{m,n}(\mathcal {B}(\mathcal {K}))\simeq \mathcal {B}(\mathcal {K}^n,\mathcal {K}^m)$:

$$\begin{aligned} X=[X_{ij}]\in M_{m,n}(\mathcal {X}) \leftrightarrow X:\mathcal {K}^n \rightarrow \mathcal {K}^m;\quad v^n=[v_1,\dots , v_n]^{\textrm{T}}\mapsto \Big [\sum _j X_{ij}v_j\Big ]^{\textrm{T}}\,. \end{aligned}$$

Hence the space $M_{m,n}(\mathcal {B}(\mathcal {K}))$ is naturally equipped with the norms induced by $\mathcal {B}(\mathcal {K}^n,\mathcal {K}^m)$, i.e.

$$\begin{aligned} \Vert X\Vert _{m,n}\equiv \Vert X\Vert _{M_{m,n}(\mathcal {X})}&:=\sup \{\Vert Xv^n\Vert _{\mathcal {K}^m}|v^n\in \mathcal {K}^n, \Vert v^n\Vert _{\mathcal {K}^n}\le 1\} \\  &= \sup \left\{ \left( \sum _{i=1}^m\bigg \Vert \sum _{j=1}^nX_{ij}v_j\bigg \Vert _{\mathcal {K}}^2\right) ^{\frac{1}{2}}\Bigg |\sum _{j=1}^n\Vert v_j\Vert _{\mathcal {K}}^2=1\right\} , \end{aligned}$$

where $\Vert \cdot \Vert _{\mathcal {K}}$ denotes the norm on $\mathcal {K}$. In the case $m=n$, we write $\Vert \cdot \Vert _n:=\Vert \cdot \Vert _{n,n}$. For simplicity we will also denote $\Vert X\Vert _{\mathcal {X}}\equiv \Vert X\Vert _{M_1(\mathcal {X})}$.

#### Proposition 2.1

The family of norms defined above satisfies the following two main properties, namely for any $m,n\in \mathbb {N}$,

$$\begin{aligned} \text {(i)} \quad&\Vert FXG\Vert _m \le \Vert F\Vert _{m,n}\Vert X\Vert _n\Vert G\Vert _{n,m} \quad \forall F,G^*\in M_{m,n}(\mathbb {C}), X\in M_n(\mathcal {X}), \\ \text {(ii)} \quad&\Vert X\oplus Y\Vert _{m+n} = \max \{\Vert X\Vert _n,\Vert Y\Vert _m\} \quad \forall X\in M_n(\mathcal {X}), Y\in M_m(\mathcal {X}), \end{aligned}$$

where $$X\oplus Y= \begin{pmatrix} X &  0\\ 0 &  Y \end{pmatrix}$$, and $$FXG\equiv (F\otimes \mathbb {1}_\mathcal {K})X(G\otimes \mathbb {1}_\mathcal {K})$$.

More generally,

#### Definition 2.2

A linear space $\mathcal {X}$ with a family of norms $\Vert \cdot \Vert _{m,n}$ on $M_{m,n}(\mathcal {X})$ that satisfy the above properties (i) and (ii) is called an *(abstract) operator space*.

It turns out that the only linear spaces $\mathcal {X}$ with endowed norms $\Vert \cdot \Vert _{m,n}$ on $M_{m,n}(\mathcal {X})$ satisfying properties (i) and (ii) are closed linear subspaces of some $\mathcal {B}(\mathcal {K})$, where $\mathcal {K}$ is a (possibly infinite dimensional) Hilbert space. Hence closed linear subspaces $\mathcal {X}\subset \mathcal {B}(\mathcal {K})$ are called *(concrete) operator spaces.*

### Norms on operator-valued Schatten spaces

Next we describe the operator space structure of *operator-valued Schatten norms*, sometimes also referred to as *amalgamated* $L_p$ *norms* or *Pisier norms*. For simplicity of introduction we let $\mathcal {H}$ be a Hilbert space of dimension $d<\infty$ here. Given some operator space $\mathcal {X}$, we set

$$\begin{aligned} \mathcal {S}_\infty [\mathcal {H},\mathcal {X}]:=M_d(\mathcal {X}), \end{aligned}$$

i.e. the $d\times d$ matrices taking values in $\mathcal {X}$, with the norm $\Vert \cdot \Vert _d\equiv \Vert \cdot \Vert _{M_{d}(\mathcal {X})}$. It is an operator space since we have

$$\begin{aligned} M_{m,n}(\mathcal {S}_\infty [\mathcal {H},\mathcal {X}]) \simeq M_{md,nd}(\mathcal {X}), \end{aligned}$$

which clearly satisfy Proposition 2.1 (*i*), (*ii*). We remark here that $\mathcal {S}_\infty [\mathcal {H},\mathbb {C}]=\mathcal {S}_\infty (\mathcal {H})$ follows directly from the definition above.

The main technical tool in this work are the operator space norms of Schatten-*q*-spaces of $\mathcal {X}$-valued operators $\mathcal {S}_p[\mathcal {H},\mathcal {X}]$. Since a complete description of the operator space structure of $\mathcal {S}_p[\mathcal {H},\mathcal {X}]$ is beyond the scope of the present paper, we refer to [6, 10] for more details. For their original construction via interpolation between certain Haagerup-tensor products of row- and column-operator spaces, see [20]. Remarkably, we can omit this because we are able to define and work with these norms by only understanding the norm on $\mathcal {S}_\infty [\mathcal {H},\mathcal {X}]$ discussed above. Next, we enumerate some of their core properties.

In the case where $\mathcal {H}$ is finite dimensional, these operator-valued Schatten spaces should be thought of as linear spaces of $d\times d$ matrices valued in $\mathcal {X}$, with special norms $\Vert \cdot \Vert _{\mathcal {S}_q[\mathcal {H},\mathcal {X}]}$. These extend naturally to infinite dimensional settings. Importantly, in the case where $\mathcal {X}=\mathbb {C}$ they all reduce to the well-known Banach space of Schatten class operators [6], i.e.

$$\begin{aligned} \mathcal {S}_q[\mathcal {H},\mathbb {C}]= \mathcal {S}_q(\mathcal {H}). \end{aligned}$$

They also satisfy the following duality relation, namely

5 $$\begin{aligned} \left( \mathcal {S}_p[\mathcal {H},\mathcal {X}]\right) ^* = \mathcal {S}_{p^\prime }[\mathcal {H},\mathcal {X}^*], \end{aligned}$$

where $\frac{1}{p}+\frac{1}{p^\prime }=1$ are dual indices and $\mathcal {X}^*$ is the operator space dual of $\mathcal {X}$ [21]. Since the norms for other values of *q* are defined via interpolation between $\mathcal {S}_\infty [\mathcal {H},\mathcal {X}]$ and $\mathcal {S}_1[\mathcal {H},\mathcal {X}]$ (which can be defined via (5)) [20], they satisfy many desirable properties, including Pisier’s formula, which we take as their definition.

#### Theorem 2.3

(Pisier’s formula [20]). Let $\mathcal {H}$ be a separable Hilbert space and $\mathcal {X}$ an operator space. Then for $1\le p\le \infty$ the following variational formulas hold for any $X\in \mathcal {S}_p[\mathcal {H},\mathcal {X}]$.

$$\begin{aligned} \Vert X\Vert _{\mathcal {S}_p[\mathcal {H},\mathcal {X}]}= \inf _{\underset{X=FYG}{F,G\in \mathcal {S}_{2p}(\mathcal {H}), Y\in \mathcal {S}_\infty [\mathcal {H},\mathcal {X}]}}\Vert F\Vert _{2p}\Vert Y\Vert _{\mathcal {S}_\infty [\mathcal {H},\mathcal {X}]}\Vert G\Vert _{2p}. \end{aligned}$$

Firstly we note that it is not mandatory that *F*, *G* are square, i.e. the statement still holds when taking the infimum over *F*, *G*, *Y* s.t. $F,G^*\in \mathcal {S}_{2p}(\mathcal {K},\mathcal {H})$ and $Y\in \mathcal {S}_\infty [\mathcal {K},\mathcal {X}]$ as long as $\dim \mathcal {H}\le \dim \mathcal {K}$. The statement in the case when $\mathcal {H}=\mathcal {K}$ is however sufficient in practice. Next, we prove two important direct consequences of Pisier’s formula.

#### Corollary 2.4

1) Let $\mathcal {X}$ be an operator space. Then, the norm on $S_p[\mathcal {H},\mathcal {X}]$ is invariant under local isometries $V^*,U\in \mathcal {B}(\mathcal {H},\mathcal {K})$ satisfying $U^*U=VV^*=\mathbb {1}_\mathcal {H}$. That is for any $X\in \mathcal {S}_{p}[\mathcal {H},\mathcal {X}]$ and any such $V^*,U\in \mathcal {B}(\mathcal {H},\mathcal {K})$ it holds that

$$\begin{aligned} \Vert UXV\Vert _{S_p[\mathcal {K},\mathcal {X}]} = \Vert X\Vert _{S_p[\mathcal {H},\mathcal {X}]}. \end{aligned}$$

In particular, when $\mathcal {K}=\mathcal {H}$, this means invariance under unitaries on the first system.

2) When $\dim \mathcal {H}<\infty$ one can restrict the infimum effectively over positive semidefinite operators, and hence

$$\begin{aligned}&\Vert X\Vert _{\mathcal {S}_p[\mathcal {H},\mathcal {X}]}=\inf _{F,G\in \mathcal {S}_{2p}(\mathcal {H}), F,G\ge 0}\Vert F\Vert _{2p}\Vert G\Vert _{2p}\Vert F^{-1}XG^{-1}\Vert _{\mathcal {S}_\infty [\mathcal {H},\mathcal {X}]}\\&\quad = \inf _{\underset{\Vert F\Vert _1=\Vert G\Vert _1=1}{F,G\ge 0}}\Vert F^{-\frac{1}{2p}}XG^{-\frac{1}{2p}}\Vert _{\mathcal {S}_\infty [\mathcal {H},\mathcal {X}]}\,, \end{aligned}$$

where $F^{-1},G^{-1}$ denote the generalized (Moore-Penrose) inverses of *F*, *G*.

#### Proof

The first claim follows via isometric invariance of the Schatten norms,

$$\begin{aligned} \Vert UXV\Vert _{\mathcal {S}_p[\mathcal {K},\mathcal {X}]}&= \inf _{\underset{UXV=FYG}{F,G\in \mathcal {S}_{2p}(\mathcal {K}), Y\in \mathcal {S}_\infty [\mathcal {K},\mathcal {X}]}}\Vert F\Vert _{2p}\Vert Y\Vert _{\mathcal {S}_\infty [\mathcal {K},\mathcal {X}]}\Vert G\Vert _{2p} \\  &= \inf _{\underset{X=(U^*F)Y(GV^*)}{F,G\in \mathcal {S}_{2p}(\mathcal {K}), Y\in \mathcal {S}_\infty [\mathcal {K},\mathcal {X}]}}\Vert F\Vert _{2p}\Vert Y\Vert _{\mathcal {S}_\infty [\mathcal {K},\mathcal {X}]}\Vert G\Vert _{2p} \\  &= \inf _{\underset{X=F^\prime YG^\prime }{F^\prime ,G^{\prime *}\in \mathcal {S}_{2p}(\mathcal {K},\mathcal {H}), Y\in \mathcal {S}_\infty [\mathcal {K},\mathcal {X}]}}\Vert F^\prime \Vert _{2p}\Vert Y\Vert _{\mathcal {S}_\infty [\mathcal {K},\mathcal {X}]}\Vert G^\prime \Vert _{2p} \\&= \Vert X\Vert _{\mathcal {S}_p[\mathcal {H},\mathcal {X}]}, \end{aligned}$$

where in the third line we redefined $F^\prime =U^*F\in \mathcal {S}_{2p}(\mathcal {K},\mathcal {H})$ and used that they have identical Schatten-2*p*-norms. The fourth line follows from the comment above on non-square *F*, *G*.

To prove the second statement, we let $X\in \mathcal {S}_{p}[\mathcal {H},\mathcal {X}]$, assuming $\dim \mathcal {H}<\infty$. Then, similarly to above, observe that

$$\begin{aligned} \Vert X\Vert _{\mathcal {S}_p[\mathcal {H},\mathcal {X}]}&= \inf _{\underset{F,G,Y \text { s.t. } X=FYG}{F,G\in \mathcal {S}_{2p}(\mathcal {H}), Y\in \mathcal {S}_\infty [\mathcal {H},\mathcal {X}]}}\Vert F\Vert _{2p}\Vert Y\Vert _{\mathcal {S}_\infty [\mathcal {H},\mathcal {X}]}\Vert G\Vert _{2p} \\&= \inf _{\underset{F,G,Y \text { s.t. }X=P_F(UYV)P_G}{F,G\in \mathcal {S}_{2p}(\mathcal {H}), Y\in \mathcal {S}_\infty [\mathcal {H},\mathcal {X}]}}\Vert P_F\Vert _{2p}\Vert Y\Vert _{\mathcal {S}_\infty [\mathcal {H},\mathcal {X}]}\Vert P_G\Vert _{2p} \\&= \inf _{\underset{F,G,Y \text { s.t. }X=P_FYP_G}{F,G\in \mathcal {S}_{2p}(\mathcal {H}), Y\in \mathcal {S}_\infty [\mathcal {H},\mathcal {X}]}}\Vert P_F\Vert _{2p}\Vert Y\Vert _{\mathcal {S}_\infty [\mathcal {H},\mathcal {X}]}\Vert P_G\Vert _{2p}, \end{aligned}$$

where in the second line we set $F=P_FU$ and $G=VP_G$ to be the right-, respectively, left-polar decompositions of *F*, *G*, such that $P_F,P_G\ge 0$. For the third equality we used the above and renamed *UYV* to *Y*. Denote with $P_F^{-1},P_G^{-1}$ their Moore-Penrose inverses and with $\Pi _F:=P_FP_F^{-1}=P_F^{-1}P_F$ the projection onto the support of $P_F$, and analogously for *G*. Now for a triple (*F*, *G*, *Y*) that occurs in the infimum we define $\tilde{Y}:=\Pi _FY\Pi _G$. Then by Proposition 2.1*i*), see also [20, Lemma 1.6], it follows that

$$\begin{aligned} \Vert \Pi _FY\Pi _G\Vert _{\mathcal {S}_\infty [\mathcal {H},\mathcal {X}]} \le \Vert Y\Vert _{\mathcal {S}_\infty [\mathcal {H},\mathcal {X}]}, \end{aligned}$$

since $\Vert \Pi _F\Vert ,\Vert \Pi _G\Vert \le 1$. Hence it holds that

$$\begin{aligned} \Vert X\Vert _{\mathcal {S}_p[\mathcal {H},\mathcal {X}]} \ge \inf _{\underset{F,G,Y \text { s.t. }X=P_FYP_G}{F,G\in \mathcal {S}_{2p}(\mathcal {H}), Y\in \mathcal {S}_\infty [\mathcal {H},\mathcal {X}]}}\Vert P_F\Vert _{2p}\Vert \Pi _FY\Pi _G\Vert _{\mathcal {S}_\infty [\mathcal {H},\mathcal {X}]}\Vert P_G\Vert _{2p}. \end{aligned}$$

On the other hand for any suitable triple (*F*, *G*, *Y*) it follows that

$$\begin{aligned} X=P_FYP_G=P_F\Pi _FY\Pi _GP_G=P_F\tilde{Y}P_G, \end{aligned}$$

hence $(F,G,\Pi _FY\Pi _G)$ is also a compatible triple. Since we have by definition an injection from suitable triples (*F*, *G*, *Y*) to ones $(F,G,\Pi _FY\Pi _G)$ it follows that

$$\begin{aligned} \Vert X\Vert _{\mathcal {S}_p[\mathcal {H},\mathcal {X}]} \le \inf _{\underset{ X=P_FYP_G}{F,G\in \mathcal {S}_{2p}(\mathcal {H}), Y\in \mathcal {S}_\infty [\mathcal {H},\mathcal {X}]}}\Vert P_F\Vert _{2p}\Vert \Pi _FY\Pi _G\Vert _{\mathcal {S}_\infty [\mathcal {H},\mathcal {X}]}\Vert P_G\Vert _{2p}. \end{aligned}$$

So overall we have shown equality. Now by construction we further have

$$\begin{aligned} P_F^{-1}XP_G^{-1} = \Pi _FY\Pi _G=\tilde{Y} \end{aligned}$$

and in total we get

$$\begin{aligned} \Vert X\Vert _{\mathcal {S}_p[\mathcal {H},\mathcal {X}]}&= \inf _{P_F,P_G\in \mathcal {S}_{2p}(\mathcal {H}), P_F,P_G\ge 0}\Vert P_F\Vert _{2p}\Vert P_F^{-1}XP_G^{-1}\Vert _{\mathcal {S}_\infty [\mathcal {H},\mathcal {X}]}\Vert P_G\Vert _{2p} \\&=\inf _{\underset{\Vert F\Vert _1=\Vert G\Vert _1=1}{F,G\ge 0}}\Vert F^{-\frac{1}{2p}}XG^{-\frac{1}{2p}}\Vert _{\mathcal {S}_\infty [\mathcal {H},\mathcal {X}]}\,, \end{aligned}$$

which is what we wanted to show. $\square$

Although it is not obvious from the above, the expression in Theorem 2.3 does define a norm. In particular, it satisfies the triangle inequality, Hölder’s duality, and the property that $\Vert X\Vert _{\mathcal {S}_q[\mathcal {H},\mathcal {S}_q(\mathcal {K})]}=\Vert X\Vert _{\mathcal {S}_q(\mathcal {H}\otimes \mathcal {K})}$. This last property follows from the more general fact that for two Hilbert spaces $\mathcal {H}, \mathcal {K}$

6 $$\begin{aligned} \mathcal {S}_q[\mathcal {H},\mathcal {S}_q[\mathcal {K},\mathcal {X}]] \simeq \mathcal {S}_q[\mathcal {H}\otimes \mathcal {K},\mathcal {X}]\simeq \mathcal {S}_q[\mathcal {K},\mathcal {S}_q[\mathcal {H},\mathcal {X}]], \end{aligned}$$

where $\simeq$ means they are completely isomorphic, see Sect. 2.4, i.e. equal as operator spaces [20, Theorem 1.9].

Pisier’s Theorem 2.3 was also used to give tractable variational expressions for the norms on the spaces $\mathcal {S}_q[\mathcal {H},\mathcal {S}_p(\mathcal {K})]$, see e.g. the case $\mathcal {X}=\mathcal {S}_p[\mathcal {K},\mathbb {C}]=\mathcal {S}_p(\mathcal {K})$ [10, Section 3.5]. These make the operator-valued Schatten norms with two indices very tractable for applications in quantum information theory, see e.g. [5–8, 10, 12, 13, 27].

#### Theorem 2.5

([20]). Given an element $X\in \mathcal {S}_q[\mathcal {H}_1,\mathcal {S}_p(\mathcal {H}_2)]$ acting on the Hilbert space $\mathcal {H}_1\otimes \mathcal {H}_2$ it holds that

$$\begin{aligned} \Vert X_{12}\Vert _{\mathcal {S}_q[\mathcal {H}_1,\mathcal {S}_p(\mathcal {H}_2)]} = {\left\{ \begin{array}{ll} \inf _{\underset{X_{12}=F_1Y_{12}G_1}{F,G\in \mathcal {S}_{2r}(\mathcal {H}_1),Y\in \mathcal {S}_{p}(\mathcal {H}_{12})}} \Vert F\Vert _{2r}\Vert G\Vert _{2r}\Vert Y\Vert _{p}\,, &  \quad for q\le p \\ \Vert X_{12}\Vert _p\,, &  \quad for q=p \\ \sup _{F,G\in \mathcal {S}_{2r}(\mathcal {H}_1)} \Vert F\Vert ^{-1}_{2r}\Vert G\Vert ^{-1}_{2r}\Vert F_1X_{12}G_1\Vert _p, &  \quad for q\ge p \end{array}\right. } \end{aligned}$$

where the infimum and supremum are over $F,G\in \mathcal {S}_{2r}(\mathcal {H}_1)$ acting only on the first Hilbert space and $Y\in \mathcal {S}_q(\mathcal {H}_{12})$ with $\frac{1}{r}:=\left| \frac{1}{q}-\frac{1}{p}\right|$. Further if $X_{12}\ge 0$, then one can choose $F=G$, see [10] or [5, Proposition 3.1 (v,vi)] and [4, Proposition 5.2 iii)].

Some of their central properties are summarized in [10]. In Lemma 3.1 below, we show that one can also obtain a version of these for the more general case where $\mathcal {S}_q(\mathcal {H})$ is replaced by any other operator space $\mathcal {X}$.

#### Notation 1

(multi-index Schatten norms) We introduce the following notation to keep track of the Schatten indices occurring in the norms, their order, value, and associated quantum systems. Given an operator $X\in \mathcal {S}_q[\mathcal {H}_A,\mathcal {S}_p[\mathcal {H}_B,\mathcal {S}_r(\mathcal {H}_C)]]$ acting on the tripartite quantum system $\mathcal {H}_A\otimes \mathcal {H}_B\otimes \mathcal {H}_C$ we write

$$\begin{aligned} \Vert X\Vert _{(A:q,B:p,C:r)} \equiv \Vert X\Vert _{\mathcal {S}_q[\mathcal {H}_A,\mathcal {S}_p[\mathcal {H}_B,\mathcal {S}_r(\mathcal {H}_C)]]} \end{aligned}$$

and more generally for a suitable $X\in \mathcal {B}(\otimes _{i=1}^k\mathcal {H}_{A_i})$

$$\begin{aligned} \Vert X\Vert _{(A_1:q_1...\,A_k:q_k)} \equiv \Vert X\Vert _{\mathcal {S}_{q_1}[\mathcal {H}_{A_1}...\,\mathcal {S}_{q_k}(\mathcal {H}_{A_k})...]}. \end{aligned}$$

Likewise, for an operator $X\in \mathcal {S}_q[\mathcal {H}_A,\mathcal {S}_p[\mathcal {H}_B,\mathcal {X}]]$ we will write

$$\begin{aligned} \Vert X\Vert _{(A:q,B:p;\mathcal {X})} \equiv \Vert X\Vert _{\mathcal {S}_q[\mathcal {H}_A,\,\mathcal {S}_p[\mathcal {H}_B,\mathcal {X}]]} \end{aligned}$$

and analogously for a different number of indices. Importantly, note also that the order in which the systems appear is determined by the order of the indices in the norm, and whenever possible we will try to make the order of systems in the operator match.

That is, formally we have in this notation for $X\in \mathcal {B}(\mathcal {H}_A)$ and $Y\in \mathcal {B}(\mathcal {H}_B)$,

$$\begin{aligned} \Vert X\otimes Y\Vert _{(A:q,B:p)} = \Vert Y\otimes X\Vert _{(A:q,B:p)} = \Vert X\Vert _q\Vert Y\Vert _p\,, \end{aligned}$$

but we try to avoid the notation $\Vert Y\otimes X\Vert _{(A:q,B:p)}$ as much as possible to avoid confusions.

The following is a direct consequence of (6):

#### Proposition 2.6

Consecutive Schatten indices of the same value can be combined:

$$\begin{aligned} \Vert X\Vert _{(A_1:q_1,...,A_{m-1}:q_{m-1},A_{m}:p,...,A_{m+n}:p;\mathcal {X})} = \Vert X\Vert _{(A_1:q_1...,A_{m-1}:q_{m-1},A_{m}\cdots A_{m+n}:p;\mathcal {X})}, \end{aligned}$$

This in particular implies that if all Schatten indices are equal, then the norm

$$\begin{aligned} \Vert X\Vert _{(A_1:p...\,A_k:p)}=\Vert X\Vert _p \end{aligned}$$

reduces to the Schatten-*p*-norm of *X*.

In the next proposition, we show that operator-valued Schatten norms enjoy a natural multiplicativity property.

#### Proposition 2.7

For any $$A\otimes X\in S_q[\mathcal {H},\mathcal {X}]$$,

$$\begin{aligned} \Vert A\otimes X\Vert _{S_q[\mathcal {H},\mathcal {X}]}=\Vert A\Vert _q\Vert X\Vert _{\mathcal {X}}\,. \end{aligned}$$

More generally for $\bigotimes _{i=1}^k X_i\in \mathcal {S}_{q_1}[\mathcal {H}_{A_1}[\mathcal {S}_{q_2}[...\,\mathcal {S}_{q_k}(\mathcal {H}_{A_k})]...]]$,

$$\begin{aligned} \bigg \Vert \bigotimes _{i=1}^k X_i\bigg \Vert _{(A_1:q_1,...,A_k:q_k)}= \prod _{i=1}^k \Vert X_i\Vert _{(A_i:q_i)}. \end{aligned}$$

#### Proof

In case that $d:=\dim \mathcal {H}<\infty$ the first statement follows from Corollary 2.4:

$$\begin{aligned} \Vert A\otimes X\Vert _{S_q[\mathcal {H},\mathcal {X}]}&= \inf _{F,G\in \mathcal {S}_{2q}(\mathcal {H}), F,G\ge 0}\Vert F\Vert _{2q}\Vert G\Vert _{2q}\Vert F^{-1}AG^{-1}\otimes X\Vert _{\mathcal {S}_\infty [\mathcal {H},\mathcal {X}]} \\&=\inf _{F,G\in \mathcal {S}_{2q}(\mathcal {H}), F,G\ge 0}\Vert F\Vert _{2q}\Vert G\Vert _{2q}\Vert F^{-1}AG^{-1}\Vert _\infty \Vert X\Vert _{\mathcal {X}} \\&= \Vert A\Vert _q\Vert X\Vert _\mathcal {X}. \end{aligned}$$

Here in the second line we used that $\Vert B\otimes X\Vert _{S_\infty [\mathcal {H},\mathcal {X}]}\!=\!\Vert B\otimes X\Vert _{M_d(\mathcal {X})}\!=\!\Vert B\Vert _{M_d(\mathbb {C})}\Vert X\Vert _\mathcal {X}= \Vert B\Vert _\infty \Vert X\Vert _\mathcal {X}$, which follows from Proposition 2.1 and the definition of the $M_d(\mathcal {X})$ norm. Alternatively this also follows by assuming without loss of generality that *A* is block-diagonal, since else we may absorb SVD-unitaries into the norm by Corollary 2.4, and applying [20, Corollary 1.3]. The second statement now follows directly by induction:

$$\begin{aligned} \bigg \Vert \bigotimes _{i=1}^k X_i\bigg \Vert _{(A_1:q_1,...,A_k:q_k)}&= \bigg \Vert X_1\otimes \bigotimes _{i=2}^{k} X_i\bigg \Vert _{S_{q_1}[\mathcal {H}_{A_1},\,\mathcal {X}]} = \Vert X_1\Vert _{q_1} \bigg \Vert \bigotimes _{i=2}^k X_i\bigg \Vert _{(A_2:q_2,...,A_k:q_k)}, \end{aligned}$$

where we used $\mathcal {X}=S_{q_2}[\mathcal {H}_{A_2}...\,\mathcal {S}_{q_k}(\mathcal {H}_{A_k})]...]$. $\square$

### Norms on linear maps

For a linear map $\Phi : \mathcal {S}_{q_1}[\mathcal {H}_{A_1}...\,\mathcal {S}_{q_k}(\mathcal {H}_{A_k})...]\rightarrow \mathcal {S}_{p_1}[\mathcal {H}_{B_1}...\,$ $\mathcal {S}_{p_l}(\mathcal {H}_{B_l})...]$ we set

$$\begin{aligned} \Vert \Phi \Vert _{(A_1:q_1...\,A_k:q_k)\rightarrow (B_1:p_1...\,B_l:p_l)}:=\sup _{X\ne 0} \frac{\Vert \Phi (X)\Vert _{(B_1:p_1...\,B_l:p_l)}}{\Vert X\Vert _{(A_1:q_1...\,A_k:q_k)}}, \end{aligned}$$

where the supremum is over all $X\in \mathcal {S}_{q_1}[\mathcal {H}_{A_1}...\,\mathcal {S}_{q_k}(\mathcal {H}_{A_k})...]$. We write a $^+$ superscript next to its channel norm when we restrict the optimization over positive semidefinite operators, i.e.

$$\begin{aligned} \Vert \Phi \Vert ^+_{(A_1:q_1...\,A_k:q_k)\rightarrow (B_1:p_1...\,B_l:p_l)}:=\sup _{X\ge 0, X\ne 0} \frac{\Vert \Phi (X)\Vert _{(B_1:p_1...\,B_l:p_l)}}{\Vert X\Vert _{(A_1:q_1...\,A_k:q_k)}}. \end{aligned}$$

This is useful when taking these maps as quantum channels acting on (positive) quantum states. For most channel norms of interest in our applications it is known that for a CP map $\Phi$, one can restrict the optimization over positive elements only without changing the norm, i.e. $\Vert \Phi \Vert =\Vert \Phi \Vert ^+$, see Lemma A.2.

The *completely bounded (cb)* norm of a linear map is defined as

$$\begin{aligned}&\Vert \Phi \Vert _{cb,(A_1:q_1...\,A_k:q_k)\rightarrow (B_1:p_1...\,B_l:p_l)}\\&\quad := \sup _E\Vert {{\,\textrm{id}\,}}_E\otimes \Phi \Vert _{(E:\infty ,A_1:q_1...\,A_k:q_k)\rightarrow (E:\infty ,B_1:p_1...\,B_l:p_l)}\,, \end{aligned}$$

where the supremum is over environment systems *E* of arbitrary size. It was shown in [20, Lemma 1.7], however, that the cb-norm is independent of the index of the environment *E*,

$$\begin{aligned} \Vert \Phi \Vert _{cb,(A_1:q_1...\,A_k:q_k)\rightarrow (B_1:p_1...\,B_l:p_l)} = \sup _E\Vert {{\,\textrm{id}\,}}_E\otimes \Phi \Vert _{(E:t,A_1:q_1...\,A_k:q_k)\rightarrow (E:t,B_1:p_1...\,B_l:p_l)}, \end{aligned}$$

for any $1\le t\le \infty$. We will be choosing this index at our convenience. One immediate consequence of this is that for any linear map $\Phi :\mathcal {X}\rightarrow \mathcal {Y}$ between operator spaces it holds that

$$\begin{aligned} \Vert {{\,\textrm{id}\,}}_A\otimes \Phi \Vert _{cb,\mathcal {S}_q[\mathcal {H}_A,\mathcal {X}]\rightarrow \mathcal {S}_q[\mathcal {H}_A,\mathcal {Y}]} = \Vert \Phi \Vert _{cb,\mathcal {X}\rightarrow \mathcal {Y}} \end{aligned}$$

for any quantum system *A*.

A linear map between operator spaces is called a *complete isometry* if it is invertible and the map and its inverse both have a CB norm equal to 1. A well-known occurrence of the CB norm in quantum information theory is the diamond norm: for a difference of channels $\Phi -\Psi :A\rightarrow B$

$$\begin{aligned} \Vert \Phi -\Psi \Vert _\diamond = \sup _E\,\Vert {{\,\textrm{id}\,}}_E\otimes (\Phi -\Psi )\Vert _{(EA:1)\rightarrow (EB:1)} = \Vert \Phi -\Psi \Vert _{cb,(A:1)\rightarrow (B:1)}\,. \end{aligned}$$

Of important interest to the present discussion is the SWAP map $F_{A\leftrightarrow B}:\mathcal {B}(\mathcal {H}_A\otimes \mathcal {H}_B)\rightarrow \mathcal {B}(\mathcal {H}_B\otimes \mathcal {H}_A): X_A\otimes X_B\mapsto X_B\otimes X_A$. It is is a $\textit{complete contraction}$ when acting on $\mathcal {S}_p[\mathcal {H}_A,\mathcal {S}_q(\mathcal {H}_B)]$ for $q\ge p$ [10, Theorem 8]. This means that for $q\ge p$, any *t* and system *E*:

7 $$\begin{aligned} \Vert {{\,\textrm{id}\,}}_E\otimes F_{A \leftrightarrow B}\Vert _{(E:t,A:p,B:q)\rightarrow (E:t,B:q,A:p)} \le \Vert F_{A \leftrightarrow B}\Vert _{cb,(A:p,B:q)\rightarrow (B:q,A:p)} \le 1\,. \end{aligned}$$

This implies that, in our notations $\Vert X\Vert _{(B:q,A:p)}=\Vert X_{BA}\Vert _{(B:q,A:p)}\le \Vert X_{AB}\Vert _{(A:p,B:q)}=\Vert X\Vert _{(A:p,B:q)}$, whenever $q\ge p$. Further due to (6) it holds that for any operator space $\mathcal {X}$ and quantum systems *E*, *A*, *B*, 

8 $$\begin{aligned} \Vert {{\,\textrm{id}\,}}_E\otimes F_{A\leftrightarrow B}\otimes {{\,\textrm{id}\,}}_{\mathcal {X}}\Vert _{(E:t,A:q,B:q;\mathcal {X})\rightarrow (E:t,B:q,A:q;\mathcal {X})} = 1 \end{aligned}$$

is a complete isometry for any *q*.

The CB norm as defined above satisfies nice properties, which makes it very versatile and powerful. One of them is a very simple general chain rule, which we make use of multiple times.

#### Lemma 2.8

Let $\Psi :\mathcal {X}\rightarrow \mathcal {Y}$ and $\Phi :\mathcal {Y}\rightarrow \mathcal {Z}$ be two maps between arbitary operator spaces $\mathcal {X},\mathcal {Y},\mathcal {Z}$, then

$$\begin{aligned} \Vert \Phi \circ \Psi \Vert _{cb,\mathcal {X}\rightarrow \mathcal {Z}} \le \Vert \Psi \Vert _{cb,\mathcal {X}\rightarrow \mathcal {Y}}\cdot \Vert \Phi \Vert _{cb,\mathcal {Y}\rightarrow \mathcal {Z}} \end{aligned}$$

### Rényi conditional entropies

We emphasize an important consequence of the variational expression that makes it a powerful tool in quantum information theory. Namely that by the first variational expression in Theorem 2.5 one may express the optimized Rényi-conditional entropy as a $\Vert \cdot \Vert _{(1,\alpha )}$ norm. Recall that for $1< \alpha <\infty$ the sandwiched Rényi$-\alpha$ divergence [19, 27] between two quantum states $\rho ,\sigma \in \mathcal {D}(\mathcal {H})$ is defined as

$$\begin{aligned} D_\alpha (\rho \Vert \sigma ):= \frac{\alpha }{\alpha -1}\log \Vert \sigma ^{\frac{1-\alpha }{2\alpha }}\rho \sigma ^{\frac{1-\alpha }{2\alpha }}\Vert _\alpha \end{aligned}$$

and the optimized Rényi$-\alpha$ conditional entropy is defined as

$$\begin{aligned} H^\uparrow _\alpha (X|Y)_\rho := -\inf _{\sigma _Y\in \mathcal {D}(\mathcal {H}_Y)}D_{\alpha }(\rho _{XY}\Vert \mathbb {1}_X\otimes \sigma _Y). \end{aligned}$$

In the limit $\alpha \rightarrow 1^+$, we recover the standard conditional entropy $H_1(A|B)_\rho \equiv H(A|B)_\rho$. In [10, (3.22)], without yet calling it as such, the above was expressed as the following $(1,\alpha )$-Pisier norm,

$$\begin{aligned} H^\uparrow _\alpha (X|Y)_\rho = \frac{\alpha }{1-\alpha }\log \Vert \rho _{YX}\Vert _{(Y:1,X:\alpha )}. \end{aligned}$$

Similarly we have that, given a quantum channel (a CPTP map) $\Phi :Q\rightarrow RS$, the minimum $\alpha$-Rényi entropy of the output system *S* given *R* under this map is proportional to the logarithm of the channel $1\rightarrow (1,p)$ norm, since

$$\begin{aligned} \inf _{\rho _{Q}\in \mathcal {D}(\mathcal {H}_{Q})}H_\alpha ^\uparrow (S|R)_{\Phi (\rho )}&= \inf _{\rho \in \mathcal {D}(\mathcal {H}_{Q})}\frac{\alpha }{1-\alpha }\log \Vert \Phi (\rho )\Vert _{(R:1,S:\alpha )} \\  &= \frac{\alpha }{1-\alpha }\log \sup _{\rho \ge 0} \frac{\Vert \Phi (\rho )\Vert _{(R:1,S:\alpha )}}{\Vert \rho \Vert _{(Q:1)}} \\  &= \frac{\alpha }{1-\alpha }\log \Vert \Phi \Vert ^+_{(Q:1)\rightarrow (R:1,S:\alpha )}. \end{aligned}$$

For the $cb,1\rightarrow (1,p)$ norm we have a similar connection. Namely, its logarithm is related to the minimal conditional Rényi entropy under purifications, i.e.

$$\begin{aligned}&\inf _{E}\inf _{\rho _{EQ}}H^\uparrow _\alpha (S|RE)_{{{\,\textrm{id}\,}}_E\otimes \Phi (\rho )} \\&\quad = \inf _{\rho _{Q}}H^\uparrow _\alpha (S|R\tilde{Q})_{({{\,\textrm{id}\,}}_{\tilde{Q}}\otimes \Phi )(|\sqrt{\rho }\rangle \langle \sqrt{\rho }|_{\tilde{Q}Q})} = \frac{\alpha }{1-\alpha }\log \Vert \Phi \Vert ^+_{cb,(Q:1)\rightarrow (R:1,S:\alpha )}, \end{aligned}$$

where the infimum in both cases is over positive normalized states $\rho$ and $\tilde{Q}$ are systems isomorphic to *Q*. Here $|\sqrt{\rho }\rangle \langle \sqrt{\rho }|_{\tilde{Q}Q}$ is a purification of $\rho _Q$ with purifying system $\tilde{Q}$, e.g. $|\sqrt{\rho }\rangle :=\sum _{i}|i\rangle _{\tilde{Q}}\otimes \sqrt{\rho _Q}|i\rangle _{Q}$. This follows from a standard Schmidt decomposition argument that yields that the CB norm is achieved on environments *E* which are isomorphic to the input system *Q* of the channel, see Lemma A.1 for details.

In the next section, we derive useful variational expressions for the norms on the operator spaces $\mathcal {S}_q[\mathcal {H}_Q,\mathcal {S}_p[\mathcal {H}_P,\mathcal {S}_r(\mathcal {H}_R)]]$, i.e. for operator-valued Schatten norms of 3 indexes, departing from Theorem 2.3. These will be central to show chain rules and sub-multiplicativity of completely bounded $1\rightarrow (1,p)$ norms that will translate directly into additivity statements for conditional entropies under tensor products of quantum channels.

## Generalized Variational Expressions for Pisier Norms

In this section, we first derive generalized variational formulas for relating the norms of $\mathcal {S}_p[\mathcal {H},\mathcal {X}]$ and $\mathcal {S}_q[\mathcal {H},\mathcal {X}]$ in Lemma 3.1. By iteration, this will allow us to derive tractable formulas for the norms of multi-index Schatten norms $\mathcal {S}_{p_1}[{{\,\mathrm{\mathcal {H}}\,}}_{A_1},\mathcal {S}_{p_2}[...\,\mathcal {S}_{p_k}({{\,\mathrm{\mathcal {H}}\,}}_{A_k})\dots ]]$. In particular we derive variational formulas for systems made of three subsystems in Theorem 3.4.

Pisier’s formula Theorem 2.3 gives a way of relating the *p*-norm on the “left” most system to the $\infty$-norm on it. In the following, we refine it to relate the *p*-norm to its *q*-norm, as done in Theorem 2.5. Here, we keep $\mathcal {X}$ general, so the following result generalizes Theorem 2.5, which can be recovered in the special case $\mathcal {X}=\mathcal {S}_q(\mathcal {K})$. Although the proof closely follows that of the mentioned special case, we provide it here for completeness.

### Lemma 3.1

(Generalized variational expressions). Let $\mathcal {X}$ be an operator space. Then for $1\le p\le q\le \infty$ and $\frac{1}{r}=\frac{1}{p}-\frac{1}{q}$, the following variational formulas hold for any $X\in \mathcal {S}_p[\mathcal {H},\mathcal {X}],\mathcal {S}_q[\mathcal {H},\mathcal {X}]$ respectively:

$$\begin{aligned} \operatorname {(i)}\qquad&\Vert X\Vert _{\mathcal {S}_p[\mathcal {H},\mathcal {X}]}=\inf _{\begin{array}{c} F,G\in \mathcal {S}_{2r}(\mathcal {H}), Y\in \mathcal {S}_q[\mathcal {H}, \mathcal {X}] \\ X=FYG \end{array}} \Vert F\Vert _{2r} \Vert Y\Vert _{\mathcal {S}_q[{{\,\mathrm{\mathcal {H}}\,}},\mathcal {X}]}\Vert G\Vert _{2r}\\ \operatorname {(ii)}\qquad&\Vert X\Vert _{\mathcal {S}_q[\mathcal {H},\mathcal {X}]} = \sup _{F,G\in \mathcal {S}_{2r}(\mathcal {H})}\Vert F\Vert _{2r}^{-1}\Vert FXG\Vert _{\mathcal {S}_p[\mathcal {H},\mathcal {X}]}\Vert G\Vert _{2r}^{-1}\,. \end{aligned}$$

The power of this theorem is that it can be iterated by choosing ${{\,\mathrm{\mathcal {X}}\,}}$ itself to be composed of many subsystems with Schatten spaces with potentially different indices.

### Proof

We will prove (i) by separately showing upper and lower bounds and (ii) will then follow by duality. Note that to keep notations short and as done before, we omit writing identity operators, e.g. for $F,G\in \mathcal {S}_{2r}(\mathcal {H})$ and $Y\in \mathcal {S}_\infty [\mathcal {H},\mathcal {X}]$ we will write $FYG\equiv (F\otimes \mathbb {1})Y(G\otimes \mathbb {1})$.

(i) Let $X=FYG\in \mathcal {S}_p[\mathcal {H},\mathcal {X}]$ with $F,G\in \mathcal {S}_{2r}(\mathcal {H}),Y\in \mathcal {S}_q[\mathcal {H},X]$ and suppose $Y=HZK$ with $H,K\in \mathcal {S}_{2q}(\mathcal {H}), Z\in \mathcal {S}_\infty [\mathcal {H},{\mathcal {X}}]$, then $X=FH Z KG$ and by Hölder’s inequality $\Vert FH\Vert _{2p}\le \Vert F\Vert _{2r}\Vert H\Vert _{2q}$, since $\frac{1}{p}=\frac{1}{r}+\frac{1}{q}$, we get $FH,KG\in \mathcal {S}_{2p}(\mathcal {H})$. Hence

$$\begin{aligned} \Vert X\Vert _{\mathcal {S}_p[\mathcal {H},\mathcal {X}]}&\le \Vert FH\Vert _{2p}\Vert Z\Vert _{\mathcal {S}_\infty [\mathcal {H},\mathcal {X}]}\Vert KG\Vert _{2p} \\&\le \Vert F\Vert _{2r}\Vert H\Vert _{2q}\Vert Z\Vert _{\mathcal {S}_\infty [\mathcal {H},\mathcal {X}]}\Vert K\Vert _{2q}\Vert G\Vert _{2r}\,. \end{aligned}$$

After minimization over *H*, *K*, *Z*, we obtain

$$\begin{aligned}&\Vert X\Vert _{\mathcal {S}_p[\mathcal {H},\mathcal {X}]} \le \Vert F\Vert _{2r}\Vert Y\Vert _{\mathcal {S}_q[\mathcal {H},\mathcal {X}]}\Vert G\Vert _{2r} \implies \Vert X\Vert _{\mathcal {S}_p[\mathcal {H},\mathcal {X}]} \\&\quad \le \inf _{\begin{array}{c} F,G\in \mathcal {S}_{2r}\\ Y\in \mathcal {S}_q[\mathcal {H},\mathcal {X}] \end{array}}\Vert F\Vert _{2r}\Vert Y\Vert _{\mathcal {S}_q[\mathcal {H},\mathcal {X}]}\Vert G\Vert _{2r}\,. \end{aligned}$$

On the other hand, let $\epsilon >0$. Then there exists $Y\in \mathcal {S}_\infty [\mathcal {H},\mathcal {X}]$ and $F,G\in \mathcal {S}_{2p}(\mathcal {H})$ such that $X=FYG$ and

$$\begin{aligned} \Vert X\Vert _{\mathcal {S}_p[\mathcal {H},\mathcal {X}]}+\epsilon \ge \Vert F\Vert _{2p}\Vert Y\Vert _{\mathcal {S}_\infty [\mathcal {H},\mathcal {X}]}\Vert G\Vert _{2p}\,. \end{aligned}$$

By performing a polar decomposition of *F* and *G* and absorbing the unitaries into *Y*, which does not change the norm, we may assume *F*, *G* to be positive semidefinite and thus $F^{\frac{p}{q}},G^{\frac{p}{q}}\in \mathcal {S}_{2q}(\mathcal {H})$ and $F^{\frac{p}{r}},G^{\frac{p}{r}}\in \mathcal {S}_{2r}(\mathcal {H})$. As a result, $X = F^{\frac{p}{r}} F^{\frac{p}{q}} Y G^{\frac{p}{q}} G^{\frac{p}{r}}$. Hence

$$\begin{aligned}&\inf _{\begin{array}{c} X=HWK\\ H,K\in \mathcal {S}_{2r}(\mathcal {H}), W\in \mathcal {S}_q[\mathcal {H},\mathcal {X}], \end{array}}\Vert H\Vert _{2r}\Vert W\Vert _{\mathcal {S}_q[\mathcal {H},\mathcal {X}]}\Vert K\Vert _{2r} \\&\quad \le \Vert F^{\frac{p}{r}}\Vert _{2r}\Vert F^{\frac{p}{q}}YG^{\frac{p}{q}}\Vert _{\mathcal {S}_q[\mathcal {H},\mathcal {X}]}\Vert G^{\frac{p}{r}}\Vert _{2r} \\&\quad \le \Vert F^{\frac{p}{r}}\Vert _{2r}\Vert F^{\frac{p}{q}}\Vert _{2q}\Vert Y\Vert _{\mathcal {S}_\infty [\mathcal {H},\mathcal {X}]}\Vert G^{\frac{p}{q}}\Vert _{2q}\Vert G^{\frac{p}{r}}\Vert _{2r} \\&\quad =\Vert F\Vert _{2p}\Vert Y\Vert _{\mathcal {S}_\infty [\mathcal {H},\mathcal {X}]} \Vert G\Vert _{2p} \\&\quad = \Vert X\Vert _{\mathcal {S}_p[\mathcal {H},\mathcal {X}]}+\epsilon . \end{aligned}$$

Since $\epsilon >0$ is arbitrary the claim follows.

(ii) by the duality $\mathcal {S}_{q}[\mathcal {H},\mathcal {X}]^*=\mathcal {S}_{q^\prime }[\mathcal {H},\mathcal {X}^*]$, i.e. there is a complete isometry between these two operator spaces, where $1/q+1/q^\prime =1$ (see [6, Proposition 4.3]). For $1/p + 1/p^\prime = 1$, we have $1 \le p^\prime \le q^\prime$ and also $\frac{1}{r} = \frac{1}{p^\prime } - \frac{1}{q^\prime }$, we thus obtain

$$\begin{aligned} \Vert X\Vert _{\mathcal {S}_q[\mathcal {H},\mathcal {X}]}&=\sup _Y\frac{|\hbox {Tr}[Y^*X]|}{\Vert Y\Vert _{\mathcal {S}_{q\prime }[\mathcal {H},\mathcal {X}^*]}}\\&\overset{\operatorname {(i)}}{=} \sup _Y\sup _{ Y = FZG}\frac{|\hbox {Tr}[Y^*X]|}{\Vert F\Vert _{2r}\Vert G\Vert _{2r}\Vert Z\Vert _{\mathcal {S}_{p^\prime }[\mathcal {H},\mathcal {X}^*]}} \\  &=\sup _{F,G,Z}\frac{|\hbox {Tr}[G^*Z^*F^*X]|}{\Vert F\Vert _{2r}\Vert G\Vert _{2r}\Vert Z\Vert _{\mathcal {S}_{p^\prime }[\mathcal {H},\mathcal {X}^*]}}\\&=\sup _{F,G,Z}\frac{|\hbox {Tr}[Z^*(F^*XG^*)]|}{\Vert F\Vert _{2r}\Vert G\Vert _{2r}\Vert Z\Vert _{\mathcal {S}_{p^\prime }[\mathcal {H},\mathcal {X}^*]}}\\&=\sup _{F,G}\frac{\Vert FXG\Vert _{\mathcal {S}_p[\mathcal {H},\mathcal {X}]}}{\Vert F\Vert _{2r}\Vert G\Vert _{2r}}. \end{aligned}$$

$$\square $$

Since for our applications we are mainly interested in operator-valued Schatten norms over 3 indices, we derive explicit variational formulas for the latter from the above lemma. However, in principle the same approach leads one to explicit variational formulas for arbitrarily many different indices. This is the main technical result of this section.

### Theorem 3.2

(Variational formulas 1). Consider $X_{123}\in \mathcal {S}_p[\mathcal {H}_1,\mathcal {S}_q[\mathcal {H}_2,\mathcal {S}_s(\mathcal {H}_3)]]$. For $1\le p\le q\le s\le \infty$ with $\frac{1}{r}:=\frac{1}{p}-\frac{1}{s}$ and $\frac{1}{r^\prime }:=\frac{1}{q}-\frac{1}{s}$, it holds that

$$\begin{aligned} \Vert X\Vert _{(1:p,2:q,3:s)} = \inf _{ X=G_{12}Y_{123}F_{12}}\Vert GG^*\Vert ^{\frac{1}{2}}_{(1:r,2:r^\prime )}\Vert F^*F\Vert ^{\frac{1}{2}}_{(1:r,2:r^\prime )}\Vert Y\Vert _s\,. \end{aligned}$$

For $1\le s\le q\le p\le \infty$ with $\frac{1}{r}:=\frac{1}{s}-\frac{1}{p}$ and $\frac{1}{r^\prime }:=\frac{1}{s}-\frac{1}{q}$,

$$\begin{aligned} \Vert X\Vert _{(1:p,2:q,3:s)} = \sup _{G_{12},F_{12}}\Vert G^*G\Vert ^{-\frac{1}{2}}_{(1:r,2:r^\prime )}\Vert FF^*\Vert ^{-\frac{1}{2}}_{(1:r,2:r^\prime )}\Vert G_{12}X_{123}F_{12}\Vert _s. \end{aligned}$$

These two expressions should be thought of as generalizations of Theorem 2.5 to three subsystems, for the above specified order of indices. A direct consequence of the Theorem is that the multi-index Piser norm of tensor product operators splits on the right, as compared to Proposition 2.7:

### Corollary 3.3

Let $X\in \mathcal {S}_{p}[\mathcal {H}_1,\mathcal {S}_q[\mathcal {H}_2,\mathcal {S}_s(\mathcal {H}_3)]]$ s.t. $X=Y_{12}\otimes Z_3$, then if either $1\le p\le q\le s\le \infty ,$ or $1\le s\le q\le p\le \infty ,$ it holds that

$$\begin{aligned} \Vert Y_{12}\otimes Z_3\Vert _{(1:p,2:q,3:s)} = \Vert Y\Vert _{(1:p,2:q)}\cdot \Vert Z\Vert _s \end{aligned}$$

### Proof

The proof follows from the multiplicativity of the Schatten-*s*-norm and the fact that relating the (*p*, *q*, *s*) to the (*s*, *s*, *s*) norm can be done by only affecting the first two systems. Depending on the order of *p*, *q*, *s* we apply the corresponding variational formula from Theorem 3.2. In the case of $1\le s\le q\le p\le \infty$ we get

$$\begin{aligned} \Vert Y_{12}\otimes Z_3\Vert _{(1:p,2:q,3:s)}&= \sup _{G_{12},F_{12}}\Vert G^*G\Vert ^{-\frac{1}{2}}_{(1:r,2:r^\prime )}\Vert FF^*\Vert ^{-\frac{1}{2}}_{(1:r,2:r^\prime )}\Vert G_{12}Y_{12}F_{12}\otimes Z_3\Vert _s \\  &= \sup _{G_{12},F_{12}}\Vert G^*G\Vert ^{-\frac{1}{2}}_{(1:r,2:r^\prime )}\Vert FF^*\Vert ^{-\frac{1}{2}}_{(1:r,2:r^\prime )}\Vert G_{12}Y_{12}F_{12}\Vert _s \cdot \Vert Z_3\Vert _s \\  &= \Vert Y\Vert _{1:p,2:q}\Vert Z\Vert _s. \end{aligned}$$

In the case $1\le p\le q\le s$ we can by an argument as in Corollary 2.4 assume $G,F\ge 0$ and apply the same as above now to $\Vert G_{12}^{-1}Y_{12}F^{-1}_{12}\otimes Z_3\Vert _s=\Vert G_{12}^{-1}Y_{12}F^{-1}_{12}\Vert _{s}\cdot \Vert Z_3\Vert _s$. $\square$

In a similar fashion to Theorem 3.2 we can also generalize Pisier’s formula and [10, Equation (3.3)]. We do this in the following.

### Theorem 3.4

(Variational Formulas 2). Consider $X_{123}\in \mathcal {S}_p[\mathcal {H}_1,\mathcal {S}_q[\mathcal {H}_2,\mathcal {X}]]$ for any operator space $\mathcal {X}$, then for any $1\le p,q\le \infty$ it holds that

$$\begin{aligned} \Vert X\Vert _{(1:p,2:q;\mathcal {X})}\le \inf _{ X=G_{12}Z_{123}F_{12}}\Vert GG^*\Vert ^{\frac{1}{2}}_{(1:p,2:q)}\Vert F^*F\Vert ^{\frac{1}{2}}_{(1:p,2:q)}\Vert Z\Vert _{(1:\infty ,2:\infty ;\mathcal {X})}, \end{aligned}$$

where $(1:q,2:p;\mathcal {X})$ is a shorthand for the norm on $\mathcal {S}_q[\mathcal {H}_1,\mathcal {S}_p[\mathcal {H}_2,\mathcal {X}]]$. Further for $1\le p\le q\le \infty$ equality holds.

### Proof of Theorem 3.2

The proof consists of repeated applications of Lemma 3.1 combined with simplifications due to Theorem 2.5. The first formula follows from Lemma 3.1 (i), first with $\mathcal {X} = \mathcal {S}_q[\mathcal {H}_2,\mathcal {S}_s(\mathcal {H}_3)]$ and then with $\mathcal {X} = \mathcal {S}_s(\mathcal {H}_3)$:

$$\begin{aligned} \Vert X\Vert _{(1:p,2:q,3:s)}&= \inf _{X_{123} =F_1Y_{123}G_1} \Vert F\Vert _{2\alpha }\Vert G\Vert _{2\alpha }\Vert Y\Vert _{(1:q,2:q,3:s)} \nonumber \\&= \inf _{X_{123} =F_1Y_{123}G_1} \Vert F\Vert _{2\alpha }\Vert G\Vert _{2\alpha }\Vert Y\Vert _{(12:q,3:s)} \\  &= \inf _{ X_{123}=F_1 H_{12} Z_{123} K_{12} G_1} \Vert F\Vert _{2\alpha } \Vert G\Vert _{2\alpha } \Vert H\Vert _{2r^\prime } \Vert K\Vert _{2r^\prime } \Vert Z\Vert _s \\  &= \inf _{\begin{array}{c} X_{123}=F_1H_{12}Z_{123}K_{12}G_1\\ F_1, G_1\ge 0,H_{12},K_{12} \end{array}} \Vert F\Vert _{2\alpha } \Vert G\Vert _{2\alpha } \Vert HH^*\Vert ^{\frac{1}{2}}_{r^\prime } \Vert K^*K\Vert ^{\frac{1}{2}}_{r^\prime } \Vert Z\Vert _s \\  &= \inf _{\begin{array}{c} X_{123}=M_{12} Z_{123} N_{12}\\ F_1,G_1\ge 0 \end{array}} \Vert F\Vert _{2\alpha } \Vert G\Vert _{2\alpha } \Vert F_1^{-1}M_{12}M_{12}^*F_1^{-1}\Vert ^{\frac{1}{2}}_{r^\prime }\\&\quad \Vert G_1^{-1}N_{12}^*N_{12}G_1^{-1}\Vert ^{\frac{1}{2}}_{r^\prime } \Vert Z\Vert _s \\  &= \inf _{ X_{123}=M_{12}Z_{123}N_{12}}\Vert M_{12}M_{12}^*\Vert ^{\frac{1}{2}}_{(1:r,2:r^\prime )}\Vert N_{12}^*N_{12}\Vert ^{\frac{1}{2}}_{(1:r,2:r^\prime )}\Vert Z\Vert _s \end{aligned}$$

where in the fourth line we restricted to positive *F*, *G* by polar decomposition, absorbed unitaries into *H*, *K* respectively, and set $M\equiv FH$, $N\equiv KG$ and thus $H=F^{-1}M$, $K=NG^{-1}$, where these inverses respectively are the generalized Moore-Penrose inverses of *F*, *G*. There we also used that the Schatten norms of $H^*H$ and $HH^*$ are equal, since their non-zero singular values, which are equal to their eigenvalues, are equal. In the last line, we used the expression of $\Vert \cdot \Vert _{(1:r,2:r^\prime )}$ given in Theorem 2.5 and the fact that $\frac{1}{\alpha }=\frac{1}{r}-\frac{1}{r^\prime }=\frac{1}{p}-\frac{1}{q}$. We recall that since $M_{12}M_{12}^*$ and $N^*_{12}N_{12}$ are positive, their 2-indexed Schatten norms are can be written by optimizations over just one matrix-variable *F*, *G*, respectively.

The second formula follows analogously after applying Lemma 3.1 (ii). We define $\frac{1}{\alpha }=\frac{1}{q}-\frac{1}{p}$.

$$\begin{aligned}&\Vert X\Vert _{(1:p,2:q,3:s)} \\&\qquad = \sup _{F_1,G_1} \Vert F\Vert ^{-1}_{2\alpha }\Vert G\Vert ^{-1}_{2\alpha }\Vert F_1 X_{123}G_1\Vert _{(1:q,2:q,3:s)} \\&\qquad =\sup _{\begin{array}{c} 0\le F_1,G_1\\ H_{12},K_{12} \end{array}}\Vert F\Vert ^{-1}_{2\alpha }\Vert G\Vert ^{-1}_{2\alpha }\Vert H\Vert ^{-1}_{2r^\prime }\Vert K\Vert ^{-1}_{2r^\prime } \Vert H_{12}F_1X_{123}G_1K_{12}\Vert _s \\&\qquad = \sup _{\begin{array}{c} 0\le F_1,G_1\\ M_{12},N_{12} \end{array}}\Vert F\Vert ^{-1}_{2\alpha }\Vert G\Vert ^{-1}_{2\alpha }\Vert F_1^{-1}M_{12}^*M_{12}F_1^{-1}\Vert ^{-\frac{1}{2}}_{2r^\prime }\Vert G_1^{-1}N_{12}N^*_{12}G_1^{-1}\Vert ^{-\frac{1}{2}}_{r^\prime } \Vert M_{12}X_{123}N_{12}\Vert _s \\&\qquad =\!\!\!\sup _{ M_{12},N_{12}}\!\!\!\left( \inf _{0<F_1}\!\!\!\Vert F\Vert ^2_{2\alpha }\Vert F_1^{-1}M_{12}^*M_{12}F_1^{-1}\Vert _{r^\prime }\!\!\right) ^{\!\!\!-\frac{1}{2}}\\&\quad \!\!\left( \inf _{0<G_1}\!\!\! \Vert G\Vert ^2_{2\alpha }\Vert G_1^{-1}N_{12}N_{12}^*G^{-1}_1\Vert _{r^\prime }\!\!\right) ^{\!\!\!\!-\frac{1}{2}} \, \!\!\Vert M_{12}X_{123}N_{12}\Vert _s \\&\qquad = \sup _{M_{12},N_{12}}\Vert M^*M\Vert ^{-\frac{1}{2}}_{(1:r,2:r^\prime )}\Vert FF^*\Vert ^{-\frac{1}{2}}_{(1:r,2:r^\prime )} \Vert M_{12}X_{123}N_{12}\Vert _s. \end{aligned}$$

$$\square $$

Similarly we prove the other variational expression.

### Proof of Theorem 3.4

We split the proof into the cases $p\le q$ and $p\ge q$. In the first case the claimed formula follows analogously to the first one in Theorem 3.2, when using Theorem 2.3 instead of Lemma 3.1 (*i*). Given $\frac{1}{\alpha }=\frac{1}{p}-\frac{1}{q}$, we get

$$\begin{aligned} \Vert X\Vert _{(1:p,2:q;\mathcal {X})}&{=} \inf _{ X=F_1Y_{123}G_1} \Vert F\Vert _{2\alpha }\Vert G\Vert _{2\alpha }\Vert Y\Vert _{(1:q,2:q;\mathcal {X})} \\  &= \inf _{X=F_1H_{12}Z_{123}K_{12}G_1} \Vert F\Vert _{2\alpha } \Vert G\Vert _{2\alpha } \Vert H\Vert _{2q} \Vert K\Vert _{2q} \Vert Z\Vert _{(1:\infty ,2:\infty ;\mathcal {X})} \\  &= \inf _{ X=M_{12}Z_{123}N_{12}}\Vert MM^*\Vert ^{\frac{1}{2}}_{(1:p,2:q)}\Vert N^*N\Vert ^{\frac{1}{2}}_{(1:p,2:q)}\Vert Z\Vert _{(1:\infty ,2:\infty ;\mathcal {X})}, \end{aligned}$$

where we used Lemma 3.1 (i) in the first line above, and skipped some steps since they are identical to those in the proof of the first formula above upon replacing $r^\prime \leftrightarrow q$ and keeping the last operator space $\mathcal {X}$ instead of $\mathcal {S}_s(\mathcal {H}_3)$. The inequality in the setting $p\ge q$ follows similarly as the above. Setting $\frac{1}{r}=\frac{1}{q}-\frac{1}{p}$, we have

$$\begin{aligned}&\Vert X\Vert _{(1:p,2:q;\mathcal {X})} \\&\qquad = \sup _{F_1,G_1}\Vert F\Vert _{2r}^{-1}\Vert G\Vert _{2r}^{-1}\Vert F_1X_{123}G_1\Vert _{(1:q,2:q;\mathcal {X})} \\&\qquad = \sup _{F_1,G_1}\Vert F\Vert _{2r}^{-1}\Vert G\Vert _{2r}^{-1} \inf _{F_1X_{123}G_1=H_{12}Z_{123}K_{12}}\Vert H\Vert _{2q}\Vert K\Vert _{2q}\Vert Z\Vert _{(1:\infty ,2:\infty ;\mathcal {X})} \\&\qquad = \sup _{F_1,G_1\ge 0}\Vert F\Vert _{2r}^{-1}\Vert G\Vert _{2r}^{-1} \inf _{ F_1X_{123}G_1=H_{12}Z_{123}K_{12}}\Vert H\Vert _{2q}\Vert K\Vert _{2q}\Vert Z\Vert _{(1:\infty ,2:\infty ;\mathcal {X})} \\&\qquad = \sup _{F_1,G_1\ge 0}\Vert F\Vert _{2r}^{-1}\Vert G\Vert _{2r}^{-1} \inf _{ X_{123}=F^{-1}_1H_{12}Z_{123}K_{12}G_1^{-1}}\Vert HH^*\Vert ^{\frac{1}{2}}_{q}\Vert K^*K\Vert ^{\frac{1}{2}}_{q}\Vert Z\Vert _{(1:\infty ,2:\infty ;\mathcal {X})} \\&\qquad = \sup _{F_1,G_1\ge 0}\Vert F\Vert _{2r}^{-1}\Vert G\Vert _{2r}^{-1} \inf _{ X_{123}=M_{12}Z_{123}N_{12}}\!\!\!\!\Vert F_1M_{12}M^*_{12}F_1\Vert ^{\frac{1}{2}}_{q}\ \\&\quad |G_1N^*_{12}N_{12}G_1\Vert ^{\frac{1}{2}}_{q}\Vert Z\Vert _{(1:\infty ,2:\infty ;\mathcal {X})} \\  &\qquad \le \inf _{X_{123}=M_{12}Z_{123}N_{12}}\sup _{F_1\ge 0}\Vert F\Vert _{2r}^{-1}\Vert F_1M_{12}M^*_{12}F_1\Vert ^{\frac{1}{2}}_{q}\!\sup _{G_1\ge 0}\Vert G\Vert _{2r}^{-1}\ \quad \\&|G_1N^*_{12}N_{12}G_1\Vert ^{\frac{1}{2}}_{q}\!\Vert Z\Vert _{(1:\infty ,2:\infty ;\mathcal {X})} \\  &\qquad = \inf _{ X_{123}=M_{12}Z_{123}N_{12}}\Vert MM^*\Vert ^{\frac{1}{2}}_{(1:p,2:q)}\Vert N^*N\Vert ^{\frac{1}{2}}_{(1:p,2:q)}\Vert Z\Vert _{(1:\infty ,2:\infty ;\mathcal {X})}, \end{aligned}$$

where the inequality arises from switching the supremum and infimum. $\square$

Having established these variational formulas, we now turn our attention to applying them to prove our main results about the multiplicativity of certain (completely bounded) mixed operator norms under tensor products, including in particular $1\rightarrow (1,p)$ norms.

## Main Results: Chain Rules and Additivity

We now use the operator-valued Schatten- and Pisier-norms introduced in the previous sections to establish useful properties of entropic measures evaluated on a composite system. We start with a chain rule that allows decomposing the entropy of a joint system *ST* into the appropriate entropies of *S* and *T*.

### Chain rules

Our chain rule will follow from the following multiplicativity statement stating that an identity “on the right” does not affect the norm of CP maps.

#### Theorem 4.1

Let $\Phi$ be a CP map $\Phi :QP\rightarrow RS$, $\mathcal {X}$ an operator space and $1\le q\le p\le \infty ,1\le r,s\le \infty$ then

$$\begin{aligned} \Vert \Phi \otimes {{\,\textrm{id}\,}}_{\mathcal {X}}\Vert _{(Q:q,P:p;\mathcal {X})\rightarrow (R:r,S:s;\mathcal {X})} = \Vert \Phi \Vert ^+_{(Q:q,P:p)\rightarrow (R:r,S:s)}, \end{aligned}$$

where the superscript $^+$ denotes optimization over positive semidefinite operators.

This result is a generalization of [10, Lemma 5]. In fact, [10, Lemma 5] corresponds to the special case where *P* and *S* are trivial and our proof strategy closely follows the one in [10].

Before giving the proof of this result, we discuss some consequences. A first immediate one is that

$$\begin{aligned} \Vert \Phi \Vert _{(Q:q,P:p)\rightarrow (R:r,S:s)} = \Vert \Phi \Vert ^+_{(Q:q,P:p)\rightarrow (R:r,S:s)}, \end{aligned}$$

for the above specified indices. A second direct consequence are two chain rules expressed in terms of conditional Rényi entropies. The first one is

#### Corollary 4.2

(Amortized Chain rule). For any state $\rho \in \mathcal {D}(\rho _{QPT})$ on system *QPT* and any quantum channel $\Phi :QP\rightarrow RS$ we have the following chain rule for $\alpha > 1$

$$\begin{aligned} H_\alpha ^\uparrow (ST|R)_{(\Phi \otimes {{\,\textrm{id}\,}}_T)(\rho )} - H_\alpha ^\uparrow (PT|Q)_{\rho } \ge \inf _{\omega \in {{\,\mathrm{\mathcal {D}}\,}}({{\,\mathrm{\mathcal {H}}\,}}_{QP})} \left( H_\alpha ^\uparrow (S|R)_{\Phi (\omega )}-H^\uparrow _\alpha (P|Q)_\omega \right) \end{aligned}$$

The second one is

#### Corollary 4.3

(Chain rule). For any state $\rho \in \mathcal {D}({{\,\mathrm{\mathcal {H}}\,}}_{QT})$ on systems *QT* and any quantum channel $\Phi :Q\rightarrow RS$, we have the chain rule for $\alpha > 1$

9 $$\begin{aligned}&H^{\uparrow }_\alpha (ST|R)_{(\Phi \otimes {{\,\textrm{id}\,}}_T)(\rho _{QT})} - H^{\uparrow }_\alpha (T|Q)_\rho \nonumber \\&\quad \ge \inf _{\sigma \in \mathcal {D}({{\,\mathrm{\mathcal {H}}\,}}_{Q})} H^{\uparrow }_\alpha (S|R)_{\Phi (\sigma _{Q})}, \end{aligned}$$

where the inequality is saturated for density operators $\rho _{QT}=\rho _{Q}\otimes \rho _T$ where $\rho _Q$ achieves the infimum on the right-hand side expression.

#### Remark 4.4

This second chain rule (9) should be compared to the one in [17, Lemma 3.6] with the replacements $Q \rightarrow E$, $\emptyset \rightarrow R$, $S \rightarrow A'$, $T \rightarrow A$, $R \rightarrow E'$. Our chain rule is less general in the sense that the system *R* in [17, Lemma 3.6] is chosen to be trivial (this is the relevant setting for the analysis of prepare-and-measure protocols [18]), but it has some advantages/differences: firstly, it is directly for the optimized Rényi entropy, secondly, we obtain a slight improvement in that we do not need a purifying system on the right-hand side of (9), and thirdly, we have no loss in the parameter $\alpha$.

#### Proof of Corollary 4.2 and Corollary 4.3

They follow both directly from Theorem 4.1 by setting $\mathcal {X}=S_\alpha (\mathcal {H}_T)$ and applying $\frac{\alpha }{1-\alpha }\log$. The amortized one follows when setting $q=r=1, p=s=\alpha > 1$, whereas (9) by setting *P* trivial and $p=q=r=1, s=\alpha > 1$. Then the left-hand sides become, respectively, with or without the *P*

$$\begin{aligned} \inf _{\rho _{QPT}\ge 0}\left( H^{\uparrow }_\alpha (ST|R)_{(\Phi \otimes {{\,\textrm{id}\,}}_T)(\rho _{QPT})} - H^{\uparrow }_\alpha (PT|Q)_{\rho _{QT}}\right) \end{aligned}$$

and right-hand side,

$$\begin{aligned} \inf _{\sigma _{QP}\ge 0} \left( H_\alpha ^\uparrow (S|R)_{\Phi (\sigma )}-H^\uparrow _\alpha (P|Q)_\sigma \right) , \end{aligned}$$

respectively,

$$\begin{aligned} \inf _{\sigma \in \mathcal {D}(\mathcal {H}_Q)} H^{\uparrow }_\alpha (S|R)_{\Phi (\sigma _{Q})}. \end{aligned}$$

Due to positive homogeneity the suprema over $\sigma \ge 0$ and $\sigma \in {{\,\mathrm{\mathcal {D}}\,}}({{\,\mathrm{\mathcal {H}}\,}}_Q)$ are equal. $\square$

We now proceed with the proof of Theorem 4.1, which as previously mentioned closely follows [10, Lemma 5] up to using our Theorem 3.4 in place of Theorem 2.3.

#### Proof of Theorem 4.1

First of all notice that $\Vert \Phi \otimes {{\,\textrm{id}\,}}_{\mathcal {X}}\Vert _{(Q:q,P:p;\mathcal {X})\rightarrow (R:r,S:s;\mathcal {X})} \ge \Vert \Phi \Vert ^+_{(Q:q,P:p)\rightarrow (R:r,S:s)}$, since we can just restrict the supremum on the left hand side over product operators. In fact, consider $X_{QP}\otimes Y_{\mathcal {X}}$, then by Lemma 3.1 and multiplicativity of the operator-valued Schatten norms Proposition 2.7, we obtain both

$$\begin{aligned} \Vert (\Phi \otimes {{\,\textrm{id}\,}}_{\mathcal {X}})(X_{QP}\otimes Y_{\mathcal {X}})\Vert _{(R:r,S:s;\mathcal {X})}&= \Vert \Phi (X_{QP})\otimes Y_{\mathcal {X}}\Vert _{(R:r,S:s;\mathcal {X})}\\&\quad = \Vert \Phi (X_{QP})\Vert _{(R:r,S:s)}\cdot \Vert Y_{\mathcal {X}}\Vert _{\mathcal {X}}, \\ \Vert X_{QP}\otimes Y_\mathcal {X}\Vert _{(Q:q,P:p;\mathcal {X})}&= \Vert X\Vert _{(Q:q,P:p)}\cdot \Vert Y_\mathcal {X}\Vert _{\mathcal {X}}. \end{aligned}$$

Now let $\mathbb {1}_\mathcal {X}$ be the identity element of $\mathcal {X}$. To prove the non-trivial side of the inequality, inspired by [10, Proof of Lemma 5], we consider a Kraus representation of $\Phi$: $\Phi (\rho )=\sum ^\nu _{i=1}K_i \rho K_i^*$. Then for a given $\rho \in \mathcal {S}_q[\mathcal {H}_Q,\mathcal {S}_p[\mathcal {H}_P,\mathcal {X}]]$, there exist for any $\epsilon >0$, by our extension of Pisier’s formula in Theorem 3.4 for this norm with $q\le p$, operators *A*, *B*, *Y*, s.t. $\rho =(A_{QP}\otimes \mathbb {1}_\mathcal {X})Y(B_{QP}\otimes \mathbb {1}_\mathcal {X})$ and $\Vert \rho \Vert _{(Q:q,P:p;\mathcal {X})}\ge \Vert AA^*\Vert ^{\frac{1}{2}}_{(Q:q,P:p)}\Vert B^*B\Vert ^{\frac{1}{2}}_{(Q:q,P:p)}\Vert Y\Vert _{(Q:\infty ,P:\infty ;\mathcal {X})}-\epsilon$. We have

$$\begin{aligned}&(\Phi \otimes {{\,\textrm{id}\,}}_\mathcal {X})(\rho ) = \sum _{i=1}^\nu (K_i\otimes \mathbb {1}_\mathcal {X})\rho (K_i^*\otimes \mathbb {1}_\mathcal {X})\\&\quad =\sum _{i=1}^\nu (K_iA\otimes \mathbb {1}_\mathcal {X})Y(BK_i^*\otimes \mathbb {1}_\mathcal {X}) = V_A(\mathbb {1}_{\mathbb {C}^\nu }\otimes Y)V^*_B, \end{aligned}$$

where $V_A= (K_1A\otimes \mathbb {1}_\mathcal {X},K_2A\otimes \mathbb {1}_\mathcal {X},...,K_\nu A\otimes \mathbb {1}_\mathcal {X})$ is a block row-vector with blocks $K_iA\otimes \mathbb {1}_\mathcal {X}$, and $V_B^*$ a block-column vector with blocks $BK_i^*\otimes \mathbb {1}_\mathcal {X}$, where we denote with $\nu$ the number of blocks and with *N* the system in which these block live and on which $\mathbb {1}_{\mathbb {C}^\nu }$ acts. These operators $V_A, V_B$ can be embedded into the space $\mathcal {B}(\mathbb {C}^\nu \otimes \mathcal {H}_Q\otimes \mathcal {H}_P\otimes \mathcal {X},\mathbb {C}^\nu \otimes \mathcal {H}_R\otimes \mathcal {H}_S\otimes \mathcal {X})$ by padding suitably with rows of 0 operators. For $V^*_A, V^*_B$ similarly into $\mathcal {B}(\mathbb {C}^\nu \otimes \mathcal {H}_R\otimes \mathcal {H}_S\otimes \mathcal {X},\mathbb {C}^\nu \otimes \mathcal {H}_Q\otimes \mathcal {H}_P\otimes \mathcal {X})$, by padding with columns of 0 operators. Call these extended operators, respectively, $V_A^\prime , V_B^\prime , V_A^{\prime *}, V_B^{\prime *}$. We get $V^\prime _A=\sum ^{\nu }_{i,j=1}\delta _{i1}|i\rangle \langle j|_N\otimes K_jA\otimes \mathbb {1}_\mathcal {X} \in \mathcal {B}(\mathbb {C}^\nu \otimes \mathcal {H}_Q\otimes \mathcal {H}_P\otimes \mathcal {X},\mathbb {C}^\nu \otimes \mathcal {H}_R\otimes \mathcal {H}_S\otimes \mathcal {X})$. Hence it holds that $V^\prime _AV_A^{\prime *}=|1\rangle \langle 1|_N\otimes V_AV_A^*$, similarly for *B* and $V^\prime _A(\mathbb {1}_N\otimes Y)V_B^{\prime *}=|1\rangle \langle 1|_N\otimes (\Phi \otimes {{\,\textrm{id}\,}}_{{{\,\mathrm{\mathcal {X}}\,}}})(\rho )$. Now using Theorem 3.4 on the space $\mathcal {S}_r[\mathbb {C}^\nu \otimes \mathcal {H}_R,\mathcal {S}_s[\mathcal {H}_S,\mathcal {X}]]$ we get

$$\begin{aligned}&\Vert (\Phi \otimes {{\,\textrm{id}\,}}_{{{\,\mathrm{\mathcal {X}}\,}}})(\rho _{QP{{\,\mathrm{\mathcal {X}}\,}}})\Vert _{(R:r,S:s;\mathcal {X})} = \Vert |1\rangle \langle 1|_N\otimes (\Phi \otimes {{\,\textrm{id}\,}}_\mathcal {X})(\rho _{QPT})\Vert _{(N:r,R:r,S:s;\mathcal {X})} \\&\quad = \Vert V^\prime _A(\mathbb {1}_N\otimes Y)V_B^{\prime *}\Vert _{(NR:r,S:s;\mathcal {X})} \\&\quad \le \Vert V^\prime _AV_A^{\prime *}\Vert ^{\frac{1}{2}}_{(NR:r,S:s)}\Vert V^\prime _BV_B^{\prime *}\Vert ^{\frac{1}{2}}_{(NR:r,S:s)}\Vert \mathbb {1}_N\otimes Y\Vert _{(N:\infty ,Q:\infty ,P:\infty ;\mathcal {X})} \\  &\quad = \Vert \sum _iK_iAA^*K_i^*\Vert ^{\frac{1}{2}}_{(R:r,S:s)}\Vert \sum _iK_iB^*BK_i^*\Vert ^{\frac{1}{2}}_{(R:r,S:s)}\Vert Y\Vert _{(Q:\infty ,P:\infty ;\mathcal {X})} \\&\quad = \Vert \Phi (AA^*)\Vert ^{\frac{1}{2}}_{(R:r,S:s)}\Vert \Phi (B^*B)\Vert ^{\frac{1}{2}}_{(R:r,S:s)} \Vert Y\Vert _{(Q:\infty ,P:\infty ;\mathcal {X})} \\&\quad \le \Vert \Phi \Vert ^+_{(Q:q,P:p)\rightarrow (R:r,S:s)}\Vert AA^*\Vert ^{\frac{1}{2}}_{(Q:q,P:p)}\Vert B^*B\Vert ^{\frac{1}{2}}_{(Q:q,P:p)}\Vert Y\Vert _{(Q:\infty ,P:\infty ;\mathcal {X})} \\&\quad \le \Vert \Phi \Vert ^+_{(Q:q,P:p)\rightarrow (R:r,S:s)}(\Vert \rho \Vert _{(Q:q,P:p;\mathcal {X})}+\epsilon ). \end{aligned}$$

In the first and third equality, we used the multiplicativity of the operator-valued Schatten norms Proposition 2.7. In the second line, we combined systems of equal indices. Since $\epsilon$ was arbitrary the claim follows. $\square$

In the following we will extend this to completely bounded norms in the following way.

#### Corollary 4.5

Let $\Phi :Q \rightarrow RS$ be a CP map, then for any $1\le q,p\le \infty$

$$\begin{aligned} \Vert \Phi \otimes {{\,\textrm{id}\,}}_{{{\,\mathrm{\mathcal {X}}\,}}}\Vert _{cb,(Q:q,{{\,\mathrm{\mathcal {X}}\,}})\rightarrow (R:q,S:p,{{\,\mathrm{\mathcal {X}}\,}})}= \Vert \Phi \Vert ^+_{cb,Q:q\rightarrow (R:q,S:p)}. \end{aligned}$$

#### Proof

We have, using the above Theorem 4.1 that

$$\begin{aligned}&\Vert \Phi \otimes {{\,\textrm{id}\,}}_{{{\,\mathrm{\mathcal {X}}\,}}}\Vert _{cb,(Q:q;{{\,\mathrm{\mathcal {X}}\,}})\rightarrow (R:q,S:p;{{\,\mathrm{\mathcal {X}}\,}})} = \sup _E\Vert \underbrace{{{\,\textrm{id}\,}}_E\otimes \Phi }_{=:\Psi _E}\otimes {{\,\textrm{id}\,}}_{{{\,\mathrm{\mathcal {X}}\,}}}\Vert _{(E:q,Q:q;{{\,\mathrm{\mathcal {X}}\,}})\rightarrow (E:q,R:q,S:p;{{\,\mathrm{\mathcal {X}}\,}})}\\&\quad \equiv \sup _E\Vert \Psi _E\otimes {{\,\textrm{id}\,}}_{{{\,\mathrm{\mathcal {X}}\,}}}\Vert _{(EQ:q;{{\,\mathrm{\mathcal {X}}\,}})\rightarrow (ER:q,S:p;{{\,\mathrm{\mathcal {X}}\,}})} \\&\quad {=} \sup _E\Vert \Psi _E\Vert ^+_{(EQ:q)\rightarrow (ER:q,S:p)}=\sup _E\Vert {{\,\textrm{id}\,}}_E\otimes \Phi \Vert ^+_{(E:q,Q:q)\rightarrow (E:q,R:q,S:p)}\\&\quad = \Vert \Phi \Vert ^+_{cb,(Q:q)\rightarrow (R:q,S:p)}. \end{aligned}$$

$$\square $$

This result will be important in proving Theorem 4.11. It also has an interpretation as a chain rule for Rényi-entropies when fixing $q=1$. Like above applying $\frac{\alpha }{1-\alpha }\log$ to the above directly yields

#### Corollary 4.6

Let $\Phi :Q\rightarrow RS$ be a CP map For any system *E* and any state $\rho \in \mathcal {D}({{\,\mathrm{\mathcal {H}}\,}}_{E}\otimes {{\,\mathrm{\mathcal {H}}\,}}_{Q} \otimes {{\,\mathrm{\mathcal {H}}\,}}_{T})$, we have the following chain rule

$$\begin{aligned}&H^{\uparrow }_\alpha (ST|RE)_{({{\,\textrm{id}\,}}_E\otimes \Phi \otimes {{\,\textrm{id}\,}}_T)(\rho _{EQT})} - H^{\uparrow }_\alpha (T|QE)_\rho \\&\quad \ge \inf _E\inf _{\sigma \in \mathcal {D}({{\,\mathrm{\mathcal {H}}\,}}_{QE})} H^{\uparrow }_\alpha (S|RE)_{({{\,\textrm{id}\,}}_E\otimes \Phi )(\sigma _{EQ})}\,. \end{aligned}$$

### Additivity

Making use of the previously established technical Lemmas, we present a general multiplicativity result for CB norms:

#### Theorem 4.7

(Multiplicativity of ordered CB norms). Let ${{\,\mathrm{\mathcal {X}}\,}}, {{\,\mathrm{\mathcal {Y}}\,}}$ be operator spaces and $1 \le p,q \le \infty$. Let $\Phi :{{\,\mathrm{\mathcal {S}}\,}}_q(\mathcal {H}_Q)\rightarrow {{\,\mathrm{\mathcal {S}}\,}}_p(\mathcal {H}_P),\Psi :\mathcal {X}\rightarrow \mathcal {Y}$ be CP maps, then

$$\begin{aligned} \Vert \Phi \otimes \Psi \Vert _{cb,S_q[\mathcal {H}_Q,\mathcal {X}]\rightarrow S_p[\mathcal {H}_P,\mathcal {Y}]} = \Vert \Phi \Vert _{cb,(Q:q)\rightarrow (P:p)}\Vert \Psi \Vert _{cb,\mathcal {X}\rightarrow \mathcal {Y}}. \end{aligned}$$

And as a direct consequence, it holds for CP maps $\{\Phi _i:Q_i\rightarrow P_i\}$ and numbers $1\le q_i,p_i\le \infty$ that

$$\begin{aligned} \Vert \bigotimes _{i=1}^n\Phi _i\Vert _{cb,(Q_1:q_1,...,Q_n:q_n)\rightarrow (P_1:p_1,...,P_n:p_n)} = \prod _{i=1}^n\Vert \Phi _i\Vert ^+_{cb,(Q_i:q_i)\rightarrow (P_i:p_i)}. \end{aligned}$$

#### Remark 4.8

This result is a generalization of [10, Theorem 11 and Theorem 13 c)] that corresponds to the case $q=q_1 = \cdots = q_n$ and $p=p_1 = \cdots = p_n$. Note that such multiplicativity statements under tensor products do not hold in general for non-CB norm, see e.g., [10, Section 5].

#### Proof of Theorem 4.7

This proof of the upper bound follows from a combination of Lemma 2.8 and Theorem 4.1. We apply the former to the maps $\Phi \otimes {{\,\textrm{id}\,}}_{\mathcal {Y}}:\mathcal {S}_q[\mathcal {H}_Q,\mathcal {Y}]\rightarrow \mathcal {S}_p[\mathcal {H_P,\mathcal {Y}}]$ and $({{\,\textrm{id}\,}}_Q\otimes \Psi ):\mathcal {S}_q[\mathcal {H}_Q,\mathcal {X}]\rightarrow \mathcal {S}_q[\mathcal {H}_Q,\mathcal {Y}]$ to get

$$\begin{aligned}&\Vert \Phi \otimes \Psi \Vert _{cb,S_q[\mathcal {H}_Q,\mathcal {X}]\rightarrow S_p[\mathcal {H}_P,\mathcal {Y}]} \\&\quad \le \Vert \Phi \otimes {{\,\textrm{id}\,}}_\mathcal {Y}\Vert _{cb,\mathcal {S}_q[\mathcal {H}_Q,\mathcal {Y}]\rightarrow \mathcal {S}_p[\mathcal {H_P,\mathcal {Y}}]} \cdot \Vert {{\,\textrm{id}\,}}_Q\otimes \Psi \Vert _{cb,\mathcal {S}_q[\mathcal {H}_Q,\mathcal {X}]\rightarrow \mathcal {S}_q[\mathcal {H_Q,\mathcal {Y}}]} \\&\quad = \Vert \Phi \Vert ^+_{cb,Q:q\rightarrow P:p}\cdot \Vert \Psi \Vert _{cb,\mathcal {X}\rightarrow \mathcal {Y}}, \end{aligned}$$

where the last line follows from Theorem 4.1 and the absorption of the ${{\,\textrm{id}\,}}_Q$ on the left is due to the definition of the CB norm. For the other inequality, for some system $E_1, E_2$, let $X_{E_1Q} \in {{\,\mathrm{\mathcal {S}}\,}}_p[{{\,\mathrm{\mathcal {H}}\,}}_{E_1}, {{\,\mathrm{\mathcal {S}}\,}}_q({{\,\mathrm{\mathcal {H}}\,}}_{Q})]$ and $Y_{E_2 {{\,\mathrm{\mathcal {X}}\,}}} \in {{\,\mathrm{\mathcal {S}}\,}}_{p}[{{\,\mathrm{\mathcal {H}}\,}}_{E_2}, {{\,\mathrm{\mathcal {X}}\,}}]$.

Let $$E=E_1E_2$$, then

$$\begin{aligned} \Vert \Phi \otimes \Psi \Vert _{cb,S_q[\mathcal {H}_Q,\mathcal {X}]\rightarrow S_p[\mathcal {H}_P,\mathcal {Y}]}&\ge \Vert ({{\,\textrm{id}\,}}_{E_1}\otimes {{\,\textrm{id}\,}}_{E_2}\otimes \Phi \otimes \Psi )(X_{E_1Q}\otimes Y_{E_2\mathcal {X}})\Vert _{(E_1:p,E_2:p,P:p;\mathcal {Y})} \\&= \Vert ({{\,\textrm{id}\,}}_{E_1}\otimes \Phi )(X_{E_1Q})\otimes ({{\,\textrm{id}\,}}_{E_2}\otimes \Psi )( Y_{E_2\mathcal {X}})\Vert _{(E_1:p,P:p,E_2:p;\mathcal {Y})} \\  &= \Vert ({{\,\textrm{id}\,}}_{E_1}\otimes \Phi )(X_{EQ})\Vert _{(E_1:p,P:p)}\cdot \Vert ({{\,\textrm{id}\,}}_{E_2}\otimes \Psi )( Y_{E_2\mathcal {X}})\Vert _{(E_2:p;\mathcal {Y})}, \end{aligned}$$

where the last equality follows from Propostion 2.7 applied to $E_1P,E_2\mathcal {Y}$. Now taking the supremum over *X*, *Y* and $E_1,E_2$ yields the claim.

The multiplicativity result for *n* tensored CP maps follows now directly via induction. For simplicity denote with $Q^n_j:=Q_j...Q_n$ and with $q_j^n:=(q_j,...,q_n)$ and similarly for *P*, *p*. Now the above is the induction start and the step follows via

$$\begin{aligned} \bigg \Vert \bigotimes _{i=1}^n\Phi _i\bigg \Vert _{cb,(Q^n:q^n)\rightarrow (P^n:p^n)}&= \bigg \Vert \Phi _1\otimes \bigotimes _{i=2}^n\Phi _i\bigg \Vert _{cb,(Q_1:q_1,Q_2^n:q_2^n)\rightarrow (P_1:p_1,P_2^n:p_2^n)} \\&=\Vert \Phi _i\Vert _{cb,(Q_1:q_1)\rightarrow (P_1:p_1)}\cdot \bigg \Vert \bigotimes _{i=2}^n\Phi _i\bigg \Vert _{cb,(Q_2^n:q_2^n)\rightarrow (P_2^n:p_2^n)}, \end{aligned}$$

where in the second line we used the above with $\mathcal {X}=\mathcal {S}_{q_2}[...\mathcal {S}_{q_n}(\mathcal {H}_{Q_n})...]$ and $\mathcal {Y}=\mathcal {S}_{p_2}[...\mathcal {S}_{p_n}(\mathcal {H}_{P_n})...]$. $\square$

As a direct consequence, we get a special case of the generalized EAT chain rule [17] for product quantum channels.

#### Corollary 4.9

(A chain rule under product maps). Consider a CP map of product form $\Phi _{Q_1Q_2 \rightarrow RS} = \phi _{Q_1 \rightarrow R} \otimes \psi _{Q_2 \rightarrow S}$. Then we have

10 $$\begin{aligned} \Vert \Phi \otimes {{\,\textrm{id}\,}}_T\Vert _{(Q_1:1,T:\alpha ,Q_2:1)\rightarrow (R:1,S:\alpha ,T:\alpha )} \le \Vert \Phi \Vert _{cb,(Q_1:1,Q_2:1)\rightarrow (R:1,S:\alpha )} \end{aligned}$$

which implies

11 $$\begin{aligned} H^{\uparrow }_\alpha (ST|R)_{(\Phi \otimes {{\,\textrm{id}\,}}_T)(\rho _{Q_{1}Q_{2}T})} \ge H^{\uparrow }_\alpha (T|Q_1)_{\rho } + \inf _{\sigma \in \mathcal {D}({{\,\mathrm{\mathcal {H}}\,}}_{Q_1Q_2} \otimes {{\,\mathrm{\mathcal {H}}\,}}_{\tilde{Q}})} H^{\uparrow }_\alpha (S|R \tilde{Q})_{(\Phi \otimes {{\,\textrm{id}\,}}_{\tilde{Q}})(\sigma _{Q_1Q_2\tilde{Q}})}, \end{aligned}$$

where $\tilde{Q}$ is a purifying system isomorphic to *Q*.

#### Remark 4.10

This chain rule (11) is similar to the one in [17, Lemma 3.6] with the replacements $Q_1 \rightarrow E$, $Q_2 \rightarrow R$, $S \rightarrow A'$, $T \rightarrow A$, $R \rightarrow E'$. The differences are the we assume the product condition which is stronger than the non-signalling condition in [17, Lemma 3.6], but we use $H_{\alpha }^{\uparrow }$ instead of $H_{\alpha }$ and our chain rule is applicable to any $\alpha \ge 1$ and there is no loss in the parameter $\alpha$.

#### Proof

We first establish (10). By the fact that we can combine systems with the same parameter (Proposition 2.6) and that the completely bounded norm is multiplicative (Theorem 4.7), we have

$$\begin{aligned}&\Vert \Phi \otimes {{\,\textrm{id}\,}}_{T} \Vert _{(Q_1:1,T:\alpha ,Q_2:1)\rightarrow (R:1,S:\alpha ,T:\alpha )} \\&\quad = \Vert \phi _{Q_1\rightarrow R}\otimes {{\,\textrm{id}\,}}_T\otimes \psi _{Q_2\rightarrow S}\Vert _{(Q_1:1,T:\alpha ,Q_2:1)\rightarrow (R:1,S:\alpha ,T:\alpha )} \\&\quad = \Vert \phi _{Q_1\rightarrow R}\otimes {{\,\textrm{id}\,}}_T\otimes \psi _{Q_2\rightarrow S}\Vert _{(Q_1:1,T:\alpha ,Q_2:1)\rightarrow (R:1,T:\alpha ,S:\alpha )} \\&\quad \le \Vert \phi _{Q_1\rightarrow R}\otimes {{\,\textrm{id}\,}}_T\otimes \psi _{Q_2\rightarrow S}\Vert _{cb,(Q_1:1,T:\alpha ,Q_2:1)\rightarrow (R:1,T:\alpha ,S:\alpha )} \\&\quad = \Vert \phi \Vert _{cb,(Q_1:1) \rightarrow (R:1)} \Vert {{\,\textrm{id}\,}}_T\Vert _{cb,T:\alpha \rightarrow T:\alpha } \Vert \psi _{Q_2\rightarrow S}\Vert _{cb,(Q_2:1) \rightarrow (S:\alpha )} \\&\quad = \Vert \phi \otimes \psi \Vert _{cb,(Q_1:1,Q_2:1) \rightarrow (R:1,S:\alpha )} \\&\quad = \Vert \Phi \Vert _{cb,(Q_1:1,Q_2:1) \rightarrow (R:1,S:\alpha )} = \Vert \Phi \Vert _{cb,(Q_1Q_2:1) \rightarrow (R:1,S:\alpha )}^+\,. \end{aligned}$$

We now show how to deduce the chain rule (11). As before, $\frac{\alpha }{1-\alpha } \log \Vert \Phi \Vert _{cb,(Q_1:1,Q_2:1) \rightarrow (R:1,S:\alpha )} = \inf _{\sigma \in \mathcal {D}({{\,\mathrm{\mathcal {H}}\,}}_{Q_1Q_2} \otimes {{\,\mathrm{\mathcal {H}}\,}}_{\tilde{Q}})} H_{\alpha }^{\uparrow }(S|R\tilde{Q})$. Moreover, given a positive semidefinite matrix $\rho _{Q_1Q_2T}$, we have that $H_{\alpha }^{\uparrow }(T|Q_1)_{\rho } = \frac{\alpha }{1-\alpha }$ $\log \Vert \rho \Vert _{(Q_1:1,T:\alpha , Q_2:1)}$ because $\Vert \rho \Vert _{(Q_1:1,T:\alpha , Q_2:1)} = \Vert \hbox {tr}_{Q_2} \rho _{Q_1TQ_2} \Vert _{(Q_1:1,T:\alpha )}$ as proved in [10, Section 3.5]. Furthermore, $H_{\alpha }^{\uparrow }(ST|R)_{(\Phi \otimes {{\,\textrm{id}\,}}_T)(\rho )} = \frac{\alpha }{1-\alpha } \log \Vert (\Phi \otimes {{\,\textrm{id}\,}}_T)(\rho )\Vert _{(R:1,S:\alpha ,T:\alpha )}$. $\square$

Motivated by applications in Sect. 5, we now consider multiplicativity with a different order. The maps $\Phi$ and $\Psi$ have composite output systems $R_1S_1$ and $R_2S_2$ and the relevant norm on the output is a multi-index Schatten norm in the order $R_1R_2S_1S_2$. We also restrict ourselves to an index *q* for the *R* systems (the index of the input systems *Q*) and an index *p* for the *S* systems.

#### Theorem 4.11

(Multiplicativity of $q \rightarrow (q,p)-$CB-norms). Let $1 \le q,p \le \infty$. Let $\Phi :Q_1\rightarrow R_1S_1$ and $\Psi :Q_2\rightarrow R_2S_2$ be two CP maps, then writing $Q^2:=Q_1Q_2$, $R^2=R_1R_2$, and $S^2=S_1S_2$, it holds that

$$\begin{aligned} \Vert \Phi \otimes \Psi \Vert _{cb,(Q^2:q)\rightarrow (R^2:q,S^2:p)} \le \Vert \Phi \Vert ^{+}_{cb,(Q_1:q)\rightarrow ({R_1}:q,{S_1}:p)} \cdot \Vert \Psi \Vert ^{+}_{cb,(Q_2:q)\rightarrow (R_2:q,{S_2}:p)}. \end{aligned}$$

As a direct consequence, it holds for CP maps $\{\Phi _i:Q_i\rightarrow R_iS_i\}$ that

$$\begin{aligned} \bigg \Vert \bigotimes ^n_{i=1}\Phi _i\bigg \Vert _{cb,(Q^n:q)\rightarrow (R^n:q,S^n:p)}\le \prod _{i=1}^n\Vert \Phi _i\Vert _{cb,{Q_i}:q\rightarrow ({R_i}:q, {S_i}:p)}, \end{aligned}$$

where we denoted $Q^n:=Q_1...Q_n$, $R^n:=R_1...R_n, S^n:=S_1...S_n$.

#### Remark 4.12

Note firstly that this result is also a generalization of [10, Theorem 11 and Theorem 13 c)] but in a different way: we recover the upper bounds of [10, Theorem 11 and Theorem 13 c)] by letting the systems *R* be trivial. Secondly, note that due to Lemma A.2 and Corollary 4.5 the optimization in the CB norms on both sides can be restricted to positive states only.

#### Proof of Theorem 4.11

Note that we may assume that all the norms are finite, otherwise the equality clearly holds. We apply Lemma 2.8 and write $\Phi \otimes \Psi$ as a composition of four maps, since the order in the multi-index norm we are considering does not respect the tensor product structure of the maps: $\Phi \otimes \Psi .$ We write it as $(F_{R_1\leftrightarrow R_2}\otimes {{\,\textrm{id}\,}}_{S_1S_2})\circ ({{\,\textrm{id}\,}}_{R_2} \otimes \Phi \otimes {{\,\textrm{id}\,}}_{S_2}) \circ (F_{Q_1 \leftrightarrow R_2} \otimes {{\,\textrm{id}\,}}_{S_2}) \circ ({{\,\textrm{id}\,}}_{Q_1} \otimes \Psi )$. Note that we wrote the swap explicitly to emphasize the order in the multi-index Schatten norms that we use. For additional clarity, we specify the operator spaces for each map: The input operator space for $({{\,\textrm{id}\,}}_{Q_1} \otimes \Psi )$ is ${{\,\mathrm{\mathcal {X}}\,}}_1 = {{\,\mathrm{\mathcal {S}}\,}}_q[{{\,\mathrm{\mathcal {H}}\,}}_{Q_1},{{\,\mathrm{\mathcal {S}}\,}}_q({{\,\mathrm{\mathcal {H}}\,}}_{Q_2})]$ and the output is ${{\,\mathrm{\mathcal {X}}\,}}_2 = {{\,\mathrm{\mathcal {S}}\,}}_q[{{\,\mathrm{\mathcal {H}}\,}}_{Q_1}, {{\,\mathrm{\mathcal {S}}\,}}_{q}[{{\,\mathrm{\mathcal {H}}\,}}_{R_2}, {{\,\mathrm{\mathcal {S}}\,}}_{p}({{\,\mathrm{\mathcal {H}}\,}}_{S_2})]]$. The output operator space of $(F_{Q_1 \leftrightarrow R_2} \otimes {{\,\textrm{id}\,}}_{S_2})$ is ${{\,\mathrm{\mathcal {X}}\,}}_3 = {{\,\mathrm{\mathcal {S}}\,}}_q[{{\,\mathrm{\mathcal {H}}\,}}_{R_2}, {{\,\mathrm{\mathcal {S}}\,}}_{q}[{{\,\mathrm{\mathcal {H}}\,}}_{Q_1}, {{\,\mathrm{\mathcal {S}}\,}}_{p}({{\,\mathrm{\mathcal {H}}\,}}_{S_2})]]$ and the output operator space of $({{\,\textrm{id}\,}}_{R_2} \otimes \Phi \otimes {{\,\textrm{id}\,}}_{S_2})$ is ${{\,\mathrm{\mathcal {X}}\,}}_4 = {{\,\mathrm{\mathcal {S}}\,}}_q[{{\,\mathrm{\mathcal {H}}\,}}_{R_2}, {{\,\mathrm{\mathcal {S}}\,}}_{q}[{{\,\mathrm{\mathcal {H}}\,}}_{R_1}, {{\,\mathrm{\mathcal {S}}\,}}_{p}[{{\,\mathrm{\mathcal {H}}\,}}_{S_1}, {{\,\mathrm{\mathcal {S}}\,}}_{p}({{\,\mathrm{\mathcal {H}}\,}}_{S_2})]]]$. The last map now maps $\mathcal {X}_4$ into $\mathcal {X}_5:={{\,\mathrm{\mathcal {S}}\,}}_q[{{\,\mathrm{\mathcal {H}}\,}}_{R_1}, {{\,\mathrm{\mathcal {S}}\,}}_{q}[{{\,\mathrm{\mathcal {H}}\,}}_{R_2}, {{\,\mathrm{\mathcal {S}}\,}}_{p}[{{\,\mathrm{\mathcal {H}}\,}}_{S_1}, {{\,\mathrm{\mathcal {S}}\,}}_{p}({{\,\mathrm{\mathcal {H}}\,}}_{S_2})]]]$. It now remains to bound the CB norm of each one of these three maps. First, by definition of the CB norm, we have

$$\begin{aligned} \Vert ({{\,\textrm{id}\,}}_{Q_1} \otimes \Psi ) \Vert _{cb,{{\,\mathrm{\mathcal {X}}\,}}_1 \rightarrow {{\,\mathrm{\mathcal {X}}\,}}_2} = \Vert \Psi \Vert _{cb, {{\,\mathrm{\mathcal {S}}\,}}_q({{\,\mathrm{\mathcal {H}}\,}}_{Q_2}) \rightarrow {{\,\mathrm{\mathcal {S}}\,}}_{q}[{{\,\mathrm{\mathcal {H}}\,}}_{R_2}, {{\,\mathrm{\mathcal {S}}\,}}_{p}({{\,\mathrm{\mathcal {H}}\,}}_{S_2})]}. \end{aligned}$$

Second, using the fact that the swap between systems of equal Schatten indices is a complete isometry and its CB norm is not affected by an identity on the right (8) gives

$$\begin{aligned} \Vert F_{Q_1 \leftrightarrow R_2} \otimes {{\,\textrm{id}\,}}_{S_2} \Vert _{cb, {{\,\mathrm{\mathcal {X}}\,}}_2 \rightarrow {{\,\mathrm{\mathcal {X}}\,}}_3}&= 1, \\ \Vert F_{R_1\leftrightarrow R_2}\otimes {{\,\textrm{id}\,}}_{S_1S_2}\Vert _{cb,\mathcal {X}_4\rightarrow \mathcal {X}_5}&= 1. \end{aligned}$$

Third, using Corollary 4.5 and also the definition of the completely bounded norm

$$\begin{aligned} \Vert {{\,\textrm{id}\,}}_{R_2} \otimes \Phi \otimes {{\,\textrm{id}\,}}_{S_2} \Vert _{cb,{{\,\mathrm{\mathcal {X}}\,}}_3 \rightarrow {{\,\mathrm{\mathcal {X}}\,}}_4} = \Vert \Phi \Vert _{cb,{{\,\mathrm{\mathcal {S}}\,}}_q({{\,\mathrm{\mathcal {H}}\,}}_{Q_1}) \rightarrow {{\,\mathrm{\mathcal {S}}\,}}_{q}[{{\,\mathrm{\mathcal {H}}\,}}_{R_1},{{\,\mathrm{\mathcal {S}}\,}}_p({{\,\mathrm{\mathcal {H}}\,}}_{S_1})]}. \end{aligned}$$

Finally, we get

12 $$\begin{aligned}&\Vert \Phi \otimes \Psi \Vert _{cb,(Q_1:q,Q_2:q)\rightarrow (R_1:q,R_2:q,S_1:p,S_2:p)} \nonumber \\&\quad \le \Vert \Phi \Vert _{cb,(Q_1:q)\rightarrow (R_1:q,S_1:p)} \cdot \Vert \Psi \Vert _{cb,(Q_2:q)\rightarrow (R_2:q,S_2:p)}. \end{aligned}$$

The proof of the *n*-fold statement follows similarly to in Theorem 4.7 by induction over the multiplicativity statement. Denote with $Q^j:=Q_1...Q_j$ for $1\le j\le n$, and analogously $R^j,S^j$. The theorem is the induction start, for the induction step observe

$$\begin{aligned} \bigg \Vert \bigotimes ^n_{i=1}\Phi _i\bigg \Vert _{cb,(Q:q)\rightarrow (R:q,S:p)}&= \left\| \bigotimes ^{n-1}_{i=1}\Phi _i\otimes \Phi _n\right\| _{cb,({Q^{n-1}:q},{Q_n}:q)\rightarrow (R^{n-1}:q,{R_n}:q,S^{n-1}:p,S_n:p)} \\  &\le \bigg \Vert \bigotimes ^{n-1}_{i=1}\Phi _i\bigg \Vert _{cb,(Q^{n-1}:q)\rightarrow (R^{n-1}:q,S^{n-1}:p)}\\&\cdot \big \Vert \Phi _n\big \Vert _{cb,(Q_n:q)\rightarrow (R_n:q,S_n:p)}, \end{aligned}$$

where the inequality in the second line follows from (12). $\square$

We also obtain a submultiplicativity result for the sequential composition of CP maps.

#### Theorem 4.13

(Sequential composition of maps). Let $\Phi :Q_1\rightarrow R_1Q_2S_1, \Psi :Q_2\rightarrow R_2S_2$ be two CP maps and write $R^2:=R_1R_2$, $S^2:=S_1S_2$, then for any $1\le q,r,s\le \infty$

$$\begin{aligned} \Vert \Psi \circ \Phi \Vert _{cb,(Q_1:q)\rightarrow (R^2:r,S^2:s)}&\le \Vert \Psi \Vert ^{+}_{cb,(Q_2:q)\rightarrow (R_2:r,S_2:s)} \cdot \Vert \Phi \Vert ^{+}_{cb,(Q_1:q)\rightarrow (R_1:r,Q_2:q,S_1:s)}. \end{aligned}$$

#### Proof

Using the fact that consecutive systems with the same Schatten index can be swapped (Proposition 2.6), we can write $\Vert \Psi \circ \Phi \Vert _{cb,(Q_1:q)\rightarrow (R^2:r,S^2:s)} = \Vert \Psi \circ \Phi \Vert _{cb,(Q_1:q)\rightarrow (R_1:r,R_2:r,S_2:s,S_1:s)}$. We then write the composition more explicitly including identities: $\Psi \circ \Phi = ({{\,\textrm{id}\,}}_{R_1} \otimes \Psi \otimes {{\,\textrm{id}\,}}_{S_1}) \circ \Phi$ and then use Lemma 2.8 to get

$$\begin{aligned}&\Vert \Psi \circ \Phi \Vert _{cb,(Q_1:q)\rightarrow (R_1:r,R_2:r,S^2:s,S_1:s)} \\&\le \Vert {{\,\textrm{id}\,}}_{R_1} \otimes \Psi \otimes {{\,\textrm{id}\,}}_{S_1} \Vert _{cb,(R_1:r,Q_2:q,S_1:s) \rightarrow (R_1:r,R_2:r,S_2:s,S_1:s)} \Vert \Phi \Vert _{cb,(Q_1:q) \rightarrow (R_1:r,Q_2:q,S_1:s)}. \end{aligned}$$

Since $\Phi$ is CP, by Lemma A.2 we have $\Vert \Phi \Vert _{cb,(Q_1:q) \rightarrow (R_1:r,Q_2:q,S_1:s)} = \Vert \Phi \Vert _{cb,(Q_1:q) \rightarrow (R_1:r,Q_2:q,S_1:s)}^+$. Then by definition of the CB norm and then using Corollary 4.5, we have

$$\begin{aligned}&\Vert {{\,\textrm{id}\,}}_{R_1} \otimes \Psi \otimes {{\,\textrm{id}\,}}_{S_1} \Vert _{cb,(R_1:r,Q_2:q,S_1:s) \rightarrow (R_1:r,R_2:r,S_2:s,S_1:s)}\\&= \Vert \Psi \otimes {{\,\textrm{id}\,}}_{S_1} \Vert _{cb,(Q_2:q,S_1:s) \rightarrow (R_2:r,S_2:s,S_1:s)} \\&= \Vert \Psi \Vert ^+_{cb,(Q_2:q) \rightarrow (R_2:r,S_2:s)}\,, \end{aligned}$$

which proves the desired result. $\square$

### Linear constraint setting and additivity

For the applications in quantum cryptography that we present in the next section, it is important to be able to restrict the optimization with a linear constraint. So we consider Theorem 4.11 for $q = 1$ by restricting the optimization implicit in the CB-norms to states satisfying some linear constraints. To enforce the linear constraints, we introduce the following definitions.

#### Definition 4.14

*(Restricted state spaces).* Let $\mathcal {H}_Q$ be the Hilbert space of a quantum system. Then we specify a linear constraint on $\mathcal {D}(\mathcal {H}_Q)$ by a linear CPTP map $\mathcal {N}:Q\rightarrow Q^\prime$ and a state $\tau \in \mathcal {D}(\mathcal {H}_{Q^\prime })$, where $Q^\prime$ is some other quantum system. Given such a tuple $r=(\mathcal {N},\tau )$ we define

$$\begin{aligned} \mathcal {D}_r({{\,\mathrm{\mathcal {H}}\,}}_Q):= \{\rho \in {{\,\textrm{Pos}\,}}(Q)|\mathcal {N}(\rho ) = \tau \hbox {Tr}[\rho ]\}. \end{aligned}$$

More generally, let $\{\mathcal {D}_{r_i}({{\,\mathrm{\mathcal {H}}\,}}_{Q_i})\}_{i\le n}$ be *n* such restricted sets of states defined in terms of the tuples $r^n=\{r_i\}_{i=1}^n=\{(\mathcal {N}_i,\tau _i)\}^n_{i=1}$ and *E* any other quantum system. Then we define

13 $$\begin{aligned} \mathcal {D}^E_{r^n}({{\,\mathrm{\mathcal {H}}\,}}_{Q^n})= \left\{ \rho \in {{\,\textrm{Pos}\,}}(EQ^n)|\textstyle \left( \bigotimes _{i\le n}\mathcal {N}_i\right) \circ \hbox {tr}_E[\rho ] = \textstyle \bigotimes _{i\le n}\tau _i\hbox {Tr}[\rho ]\right\} . \end{aligned}$$

In particular, when $\mathcal {N}:Q\rightarrow \mathbb {C}, \rho \mapsto \hbox {Tr}[\rho ]$ is the full trace and $\tau = 1$, then $\mathcal {D}_r({{\,\mathrm{\mathcal {H}}\,}}_Q) = \mathcal {D}({{\,\mathrm{\mathcal {H}}\,}}_Q)$ and $\mathcal {D}^E_r({{\,\mathrm{\mathcal {H}}\,}}_Q) = \mathcal {D}({{\,\mathrm{\mathcal {H}}\,}}_{EQ})$ is just the set of unrestricted normalized states. The restricted CB-norms of quantum channels are then naturally defined as follows.

#### Definition 4.15

*(Restricted CB-norms).* Let $r:=(\mathcal {N},\tau )$ define a linear restriction and $\Phi :Q\rightarrow RS$ be a CP map, then we define the *restricted completely bounded norm* of $\Phi$ as

$$\begin{aligned} \Vert \Phi \Vert _{r,cb,(Q:q)\rightarrow (R:r,S:s)}:=\sup _E\sup _{\rho \in \mathcal {D}_r^E({{\,\mathrm{\mathcal {H}}\,}}_Q)}\frac{\Vert ({{\,\textrm{id}\,}}_E\otimes \Phi )(\rho )\Vert _{(E:q,R:r,S:s)}}{\Vert \rho \Vert _{(E:q,Q:q)}}. \end{aligned}$$

Now, we may strengthen Theorem 4.11 to also hold under arbitrary linear constraints of the form (13).

#### Theorem 4.16

(Multiplicativity of restricted CB $1\rightarrow (1,p)$ norms). Let *n* CP maps $\Phi _i:Q_i\rightarrow R_iS_i$ such that $\Vert \Phi _i\Vert _{cb,(Q_i:1)\rightarrow (R_i:1,S_i:p)}<\infty$ and *n* linear restrictions governed by triples $r_i:=(Q_i^\prime ,\mathcal {N}_i,\tau _i)$ be given. Denote the combined spaces $Q^n:=Q_1...Q_n$, $R^n:=R_1...R_n$, and $S^n:=S_1...S_n$ and the combined linear restriction as $r^n:=(\otimes _{i=1}^n\mathcal {N}_i,\otimes _{i=1}^n\tau _i)$. Then it holds that

14 $$\begin{aligned} \bigg \Vert \bigotimes _{i=1}^n \Phi _i\bigg \Vert _{r^n,cb,(Q^n:1)\rightarrow (R^n:1,S^n:p)}&\le \prod ^n_{i=1} \Vert \Phi _i\Vert _{r_i,cb,(Q_i:1)\rightarrow (R_i:1,S_i:p)}\,. \end{aligned}$$

#### Remark 4.17

This is a generalization of a result from [25], in which this statement, formulated in terms of Rényi entropies and derived via very different tools, was shown to hold in the special case where all channels $\Phi _i=\mathcal {M}$ are equal.

#### Proof

The proof follows via lifting Theorem 4.11 to the restricted setting, in the same manner as was done in [25]. Hence it is repeated in Appendix B. $\square$

## Applications to Quantum Cryptography

We now show how the multiplicativity of restricted CB $1\rightarrow (1,p)$ norms shown in Theorem 4.16 can be used to prove the security of time-adaptive quantum cryptographic protocols. In this section we will assume all Hilbert spaces to be finite dimensional.

### *f*-weighted Rényi entropy

A key quantity used in security proofs is the *f*-weighted Rényi entropy [25], which is defined as follows. Let $\mathbb {X}$ be a finite set, $f:\mathbb {X}\rightarrow \mathbb {R}$ a function, and $\rho _{XAE} = \sum _x |{x}\rangle \langle {x}|_X \otimes \rho _{AE}^x$ a state that is classical on system *X*, then for all $\alpha >1$, define the *f*-weighted Renyi entropy as

$$\begin{aligned} H_\alpha ^{\uparrow ,f}(A|XE)_{\rho }&:= \frac{\alpha }{1-\alpha } \log \Vert 2^{\tfrac{\alpha -1}{\alpha }f_X} \cdot \rho _{EXA}\Vert _{(EX:1,A:\alpha )}\\&= \frac{\alpha }{1-\alpha } \log \sum _{x\in \mathbb {X}} 2^{\tfrac{\alpha -1}{\alpha }f(x)} \Vert \rho _{EA}^x\Vert _{(E:1,A:\alpha )} \end{aligned}$$

where $f_X = \sum _x f(x) |{x}\rangle \langle {x}|$ is a diagonal operator, which commutes with $\rho _{EXA}$. In particular, we note that when $f(x)=0$ for all $x\in \mathbb {X}$, we recover the usual Rényi entropy $H_\alpha ^{\uparrow ,f}(A|XE)_{\rho } = H_\alpha ^{\uparrow }(A|XE)_{\rho }$.

As we will see now, Theorem 4.16 implies a chain rule for *f*-weighted Rényi entropies. Assume we need to minimize the *f*-weighted Rényi entropy over a set of states of the form

$$\begin{aligned} \{ \rho _{EX^nA^n} = ({{\,\textrm{id}\,}}_E\otimes \mathcal {M}^n) (\rho _{EQ^n}) \, | \, \rho _{EQ^n} \in \mathcal {D}_r^E({{\,\mathrm{\mathcal {H}}\,}}_{Q^n})\} \end{aligned}$$

for all possible choices of environments *E*, where $\mathcal {M}^n = \bigotimes _{i=1}^n \mathcal {M}_i$ and $\mathcal {M}_i:Q_i\rightarrow X_iA_i$ are CP map and $\mathcal {D}^E_{r^n}({{\,\mathrm{\mathcal {H}}\,}}_{Q^n})$ is an environment embedded restricted state space as defined in (13). Assume moreover that $f^n(x^n)= \sum _i f_i(x_i)$. Then the results below shows that the minimium is obtained for an tensor product state of the form $\rho _{{E^\prime }^nX^nA^n} = \bigotimes _{i=1}^n \rho _{E^\prime XA}^i$ with $\rho _{E^\prime XA}^i = {{\,\textrm{id}\,}}_{E^\prime }\otimes \mathcal {M}_i(\rho ^i_{E^\prime Q})$ and $\rho _{QE'}^i\in D_r^{E'}({{\,\mathrm{\mathcal {H}}\,}}_{Q_i})$ and $E = {E'}^n$, in which case then entropy is additive.

#### Corollary 5.1

(Reduction to independent attacks). For all $i\in [n]$, let $\mathcal {M}_i:Q_i\rightarrow X_iA_i$ be a CP map between finite dimensional systems, with $X_i$ a classical register with basis elements labeled $\mathbb {X}$, and let $f_i: \mathbb {X} \rightarrow \mathbb {R}$. Set $Q^n:=Q_1...Q_n, X^n:=X_1...X_n,$ and $A^n:=A_1...A_n$ and define $\mathcal {M}^n = \bigotimes _{i=1}^n \mathcal {M}_i$ and $f^n(x^n) = \sum _{i=1}^n f_i(x_i)$. Then for all $\alpha >1$

$$\begin{aligned}&\inf _E\inf _{\rho \in \mathcal {D}^E_r({{\,\mathrm{\mathcal {H}}\,}}_{Q^n})} H_\alpha ^{\uparrow ,f_n}(A^n|X^nE)_{({{\,\textrm{id}\,}}_E\otimes \mathcal {M}^n)(\rho )}\\&= \sum _{i=1}^n \inf _E\inf _{\rho _i\in \mathcal {D}_r^E({{\,\mathrm{\mathcal {H}}\,}}_{Q_i})} H_\alpha ^{\uparrow ,f_i}(A_i|X_iE)_{({{\,\textrm{id}\,}}_E\otimes \mathcal {M}_i)(\rho _i)} \\ \end{aligned}$$

#### Proof

Define the operators $f_{i,X_i}:= \sum _{x \in \mathbb {X}} f_i(x) |{x}\rangle$ $\langle {x}|_{X_i}$ and $f^n_{X^n} = \sum _{x^n\in \mathbb {X}^n} f^n(x^n)|{x}\rangle \langle {x}|^{n}_{X^n}$ and the maps $\Phi _i: Q_i \rightarrow X_i A_i$ defined by $\Phi _i(\rho ):= 2^{\tfrac{\alpha -1}{2\alpha }f_{i,X_i}} \mathcal {M}_i(\rho ) 2^{\tfrac{\alpha -1}{2\alpha }f_{i,X_i}}$, which are CP by construction, since $2^{\frac{\alpha -1}{2\alpha }f_{i,X_i}}$ are self-adjoint. Then, we can use Theorem 4.16 for the maps $\Phi _i$ with the replacements $A_i \rightarrow S_i$ and $X_i \rightarrow R_i$ and by applying $\frac{\alpha }{1-\alpha }\log$ to each side of the equation (14) and noting that $\bigotimes _{i=1}^n 2^{f_{i,X_i}} = 2^{f^n_{X^n}}$ we get that the LHS in upper bounded by the RHS. Equality follows from additivity of $H^\uparrow _\alpha$ under tensor products of states [24, Corollary 5.9]. $\square$

### Definition of the protocol

For simplification, we will consider a random number generation (QRNG) protocol, which are closely related to quantum key distribution (QKD) protocols. Using standard techniques, the present security proof can be easily generalized to QKD [23, 25].

An *n*-round device-dependent random number generation protocol consists of two steps. First, we generate the raw data and then determine how much secure randomness can be extracted from that data. The first step can be represented as a CPTP map

$$\begin{aligned} \mathcal {M}^n: Q^n \rightarrow X^nA^n \end{aligned}$$

from an input space $Q^n=Q_1 \dots Q_n$ to the output systems $X^n$ and $A^n$, representing respectively the public announcements and the raw key. Since they are classical variables, we can write operators on the corresponding Hilbert spaces as diagonal operators in some canonical basis, the elements of which are labeled by the finite sets $\mathbb {X},\mathbb {A}$.

**Independent rounds** We assume that our protocol varies over times, but that different rounds of the protocol, corresponding to the CPTP maps $\mathcal {M}_t: {{\,\mathrm{\mathcal {D}}\,}}({{\,\mathrm{\mathcal {H}}\,}}_{Q})\rightarrow {{\,\mathrm{\mathcal {D}}\,}}({{\,\mathrm{\mathcal {H}}\,}}_{XA})$, act independently on different inputs, i.e.

$$\begin{aligned} \mathcal {M}^n = \bigotimes _{t=1}^n \mathcal {M}_t\,. \end{aligned}$$

**Linear constraint on the input** We assume that the protocol is applied to an initial unknown input state $\rho _{EQ^n}$ entangled with an arbitrary reference system *E*, and where $\rho _{Q^n} = \hbox {Tr}_E[\rho _{EQ^n}]$ satisfies a linear constraint of the form

15 $$\begin{aligned} \bigotimes _t \mathcal {N}_t(\rho _{Q^n}) = \bigotimes _t \tau _t \end{aligned}$$

where ${{\,\mathrm{\mathcal {N}}\,}}_t: {{\,\mathrm{\mathcal {D}}\,}}({{\,\mathrm{\mathcal {H}}\,}}_Q)\rightarrow {{\,\mathrm{\mathcal {D}}\,}}({{\,\mathrm{\mathcal {H}}\,}}_{Q'})$ is a completely positive map to some system $Q'$ and $\tau _t\ge 0$ a positive semidefinite operator on $Q'$. This last condition is used in prepare-and-measure quantum key distribution protocols [28]. This corresponds to saying that $\rho _{EQ^n}\in \mathcal {D}^E_{r^n}({{\,\mathrm{\mathcal {H}}\,}}_{Q^n})$ for some restricted state space defined in (13).

### Security and rate of the QRNG protocol

**Post-processing** To complete the protocol, we need to specify how we determine the amount of randomness that can be extracted from the raw key registers $A^n$. This is done using some function $g_n:\mathbb {X}^n\rightarrow \mathbb {N}$, which will be constructed below. The protocol first evaluates $k \leftarrow g_n(x^n)$, then samples an extractor $E_s: \mathbb {A}^n \rightarrow \{0,1\}^k$ from a 2-universal family with seed *s*, and finally applies the extractor to the raw key register $A^n$ and writes the *k*-bit result in the classical register $K_{x^n}$ holding bitstrings of length $g_n(x^n)$. We write the map performing this as $\mathcal {R}^{g_n}: {{\,\mathrm{\mathcal {D}}\,}}({{\,\mathrm{\mathcal {H}}\,}}_{X^nA^n}) \rightarrow {{\,\mathrm{\mathcal {D}}\,}}(\oplus _{x^n} {{\,\mathrm{\mathcal {H}}\,}}_{K_{x^n}} \otimes {{\,\mathrm{\mathcal {H}}\,}}_{S} )$ for this map.

Note that the length of the key is itself a random variable, whose distribution depends on the input state $\rho _{EQ^n}$. Moreover, values of $x^n\in \mathbb {X}^n$ for which $g_n(x^n)=0$, correspond to the cases where the protocol aborts.

**Composable security** For a given protocol $\mathcal {M}^n$, post-processing $g_n$ and input state $\rho _{Q^nE}$ satisfying (15), let $\oplus _{x^n \in \mathbb {X}^n} \rho _{K_{x^n}SE}^{x^n} = (\mathcal {R}^{g_n}\circ {{\,\mathrm{\mathcal {M}}\,}}^n \otimes {{\,\textrm{id}\,}}_E)(\rho _{Q^nE})$ be the state obtained after applying the protocol and the post-processing. It is shown in [22] that the composable security level is given by

$$\begin{aligned} \epsilon (\mathcal {M}^n,g_n,\rho ) := \frac{1}{2} \sum _{x^n} \left\| \rho ^{x^n}_{K_{x^n}SE} - \tfrac{{{\,\textrm{id}\,}}_{K_{x^n}}}{|K_{x^n}|}\otimes \rho _{SE}^x \right\| _1\,. \end{aligned}$$

The full protocol is said to be $\epsilon$-secure if for any input state $\rho _{Q^nE}$ satisfying (15), the final state satisfies $\epsilon (\mathcal {P},g_n,\rho )\le \epsilon$.

**Asymptotic rate** A protocol not only needs to be secure, it must also be efficient. Contray to the security condition, which must hold for any input state, the efficiency of the protocol is evaluated with respect to some “honest" input state, which is known in advance. Assume we are given honest input states $\rho _{Q_t}^{\textrm{hon}}$ for $t\in \mathbb {N}$, we consider the corresponding distributions $q^{\textrm{hon}}_{X_t} = \hbox {tr}_{A} \circ \mathcal {M}_t(\rho _{Q_t}^{\textrm{hon}})$ and $q^{\textrm{hon}}_{X^n} = \otimes _t q^{\textrm{hon}}_{X_t}$. Then the average key rate is given by

$$\begin{aligned} \textrm{rate}(\mathcal {M}^n, g_n, q^{\textrm{hon}}_{X^n}) = \frac{1}{n} \mathbb {E}_{x^n \sim q^{\textrm{hon}}_{X^n}}[g_n(x^n)]\,. \end{aligned}$$

In particular we will be interested in the asymptotic limit $n\rightarrow \infty$.

### Time adaptive asymptotic key rates

For every $n\in \mathbb {N}$, we want to build a post-processing function $g_n:\mathbb {X}^n \rightarrow \mathbb {R}$, so that (a) the protocol is $\epsilon$-secure for any input state and (b) it achieves the largest possible key rate on average when applied to the state $\otimes _{t=1}^n \rho _{Q_t}^{\textrm{hon}}$. How large can the average key rate be in the asymptotic limit $n\rightarrow \infty$?

**Time-invariant protocols** This question was considered by Renner in [23], who considered the case where protocols do not vary in time, i.e. $\mathcal {M}_t = \mathcal {M}$, $\mathcal {N}_t=\mathcal {N}$, $\tau _i = \tau$, $q^{\textrm{hon}}_{X_t} = q^{\textrm{hon}}_{X}$. Then it is possible to achieve an asymptotic key rate given by the conditional von Neumann entropy, minimized over all possible states that reproduce the statistics $q_X^{\textrm{hon}}$:

16a $$\begin{aligned} h(\mathcal {M},\mathcal {N},\tau ,q^{\textrm{hon}}_X) =&\inf _{E,\rho _{QE} \in {{\,\mathrm{\mathcal {D}}\,}}({{\,\mathrm{\mathcal {H}}\,}}_{QE})}&H(A|XE)_{\mathcal {M}(\rho _{QE})} \end{aligned}$$

16b $$\begin{aligned}&\mathrm {subject\ to}&\mathcal {N}(\rho _{Q}) = \tau&\end{aligned}$$

16c $$\begin{aligned}&\hbox {tr}_A \circ \mathcal {M}(\rho _Q) = q_X^{\textrm{hon}}\,. \end{aligned}$$

In other words, for every *n* there exists a function $g_n:\mathbb {X}^n\rightarrow \mathbb {R}$, such that

$$\begin{aligned} \lim _{n\rightarrow \infty } \max _{\rho _{Q^nE}} \epsilon (\mathcal {M}^n,g_n,\rho _{Q^nE})&= 0\\ \lim _{n\rightarrow \infty } \textrm{rate}(\mathcal {M}^n, g_n, (q^{\textrm{hon}}_{X})^{\otimes n})&= h(\mathcal {M},\mathcal {N},\tau ,q^{\textrm{hon}}_X). \end{aligned}$$

**Time-dependent protocols**

We generalize this to the case of protocols that vary in time, where we show that it is possible to achieve an asymptotic rate given by

$$\begin{aligned} r_{ad} := \lim _{n \rightarrow \infty } \frac{1}{n} \sum _{t=1}^n h(\mathcal {M}_t,\mathcal {N}_t,\tau _t,q_{X_t}^{\textrm{hon}}) \,. \end{aligned}$$

To understand the advantage, we should compare this secret key rate with the one we obtain by applying a static security proof. First, note that when the protocol and the noise are static, there is no advantage in using a time-adaptive security proof. When both vary with time, the comparison cannot be made because static methods do not apply. However, we can easily make the comparison when the protocol is static, i.e., $\mathcal {M}_t = \mathcal {M}$, $\tau _t = \tau$, $\mathcal {N}_t = \mathcal {N}$, but the noise is not, i.e. $q_{X_t}$ varies with *t*.

In this case, standard proof techniques [17, 23, 25] allow one to attain the secret key rate that corresponds to the average noise distribution $\bar{q}^{\textrm{hon}}_X = \lim _{n\rightarrow \infty }\frac{1}{n} \sum _{t=1}^n q^{\textrm{hon}}_{X_t}$. In other words, they allow us to construct $g_n$ such that

$$\begin{aligned} \lim _{n\rightarrow \infty } \textrm{rate}(\mathcal {P}, g_n,q_{X^n}) = h(\mathcal {M},\mathcal {N},\tau , \overline{q}^{\textrm{hon}}_X) =: r_{na}\,, \end{aligned}$$

However, the key rate obtained using our time-adaptive method is higher. This is because the function $h(\mathcal {M}, {{\,\mathrm{\mathcal {N}}\,}}, \tau , q_X)$ is a convex function in $q_X$ in general [28] and strictly convex in most cases. In the latter case, there exists distribution $q_{X_t}^{\textrm{hon}}$ such that

$$\begin{aligned} r_{na} = h(\mathcal {M},\mathcal {N},\tau , \overline{q}^{\textrm{hon}}_X) < \frac{1}{n} \sum _{t=1}^n h(\mathcal {M},\mathcal {N},\tau ,q^{\textrm{hon}}_{X_t}) = r_{ad}\,, \end{aligned}$$

which shows that the asymptotic key rate is higher using adaptive methods.

### Security proof

We show that we can achieve the adaptive rate $r_{ad}$, under a technical assumptions on the honest distribution.

#### Theorem 5.2

(Time-adaptive protocol). Let $\mathcal {M}^n$ be a family of protocols as defined in Sect. 5.2 and let $\rho ^{\textrm{hon}}_{Q_t} \in {{\,\mathrm{\mathcal {D}}\,}}({{\,\mathrm{\mathcal {H}}\,}}_{Q_t})$ for $t \in \mathbb {N}$ be a family of quantum states such that $\{(\mathcal {M}_t,\mathcal {N}_t,\mathcal {\tau }_i,q^{\textrm{hon}}_{X_t})|t\in \mathbb {N}\}$ is a finite set and $\rho ^{\textrm{hon}}_{Q_t}> 0$ for all $t\in \mathbb {N}$. Then there exists a family of functions $g_n: \mathbb {X}^n\rightarrow \mathbb {R}$ which, for all *n*, lead to an $\epsilon _n$-secure protocol, so that $\lim _{n\rightarrow \infty } \epsilon _n = 0$ and

$$\begin{aligned} \lim _{n\rightarrow \infty } \textrm{rate}(\mathcal {P}_n, g_n,q^{\textrm{hon}}_{X^n}) = \lim _{n \rightarrow \infty } \frac{1}{n} \sum _{t=1}^n h(\mathcal {M}_t,\mathcal {N}_t,\tau _t,q_{X_t}^{\textrm{hon}})= r_{ad}\,. \end{aligned}$$

#### Proof of Theorem 5.2

We first explain how to construct the functions $g_n$. It is a standard results in QKD that $h(\mathcal {M},\mathcal {N},\tau ,q_{X})$ is a convex function in $q_X$ (see for example [28]). For each $t\in \mathbb {N}$, the assumption $\rho ^{\textrm{hon}}_{Q_t} > 0$ ensures that $q_{X_t}^{\textrm{hon}}$ is a strictly feasible distribution (i.e. the inequality constraints in the convex optimization problem (16) can be satisfied with strict inequalities). Consequently, there exists a supporting hyperplane to the graph of the function $q_X \mapsto h(\mathcal {M}_t,\mathcal {N}_t,\tau _t, q_X)$ at the point $(q^{\textrm{hon}}_{X_t},h(\mathcal {M}_t,\mathcal {N}_t,\tau _t, q^{\textrm{hon}}_{X_t}))$. Moreover, we can chose the hyperplanes to be the same for all $t\in \mathbb {N}$ such that $(\mathcal {M}_t,\mathcal {N}_t,\mathcal {\tau }_t,q^{\textrm{hon}}_{X_t})$ are the same.

Let $f_t:\mathbb {X}\rightarrow \mathbb {R}$ be a function parametrizing the hyperplanes, so that $\sum _x f_t(x) q_{X}(x) \le h(\mathcal {M}_t,\mathcal {N}_t,\tau _t,q_{X})$ for all probability distributions $q_X$, and $\sum _x f_t(x) q^{\textrm{hon}}_{X_t}(x) =h(\mathcal {M},\mathcal {N},$ $\tau ,q^{\textrm{hon}}_{X_t})$. Note that a supporting hyperplane is given by an affine function, but the constant term can always be absorbed in the coefficients $f_t(x)$ since we require the inequality to hold for normalized probability distributions satisfying $\sum _{x} q_{X}(x) = 1$. Recalling the definition of *h*, this guarantees that for every *t* and every $\rho _{EXA} = \mathcal {M}_t(\rho _{EQ})$ with $\rho _{EQ}\in {{\,\mathrm{\mathcal {D}}\,}}_r^E({{\,\mathrm{\mathcal {H}}\,}}_Q)$,

$$\begin{aligned} H(A|XE)_{\rho } \ge \mathbb {E}_{x \sim \rho _X}[f_t(x)]\,. \end{aligned}$$

We define our post-processing function by $g_n(x^n) = \max (0, \lfloor f^n(x^n) - \delta (\epsilon ,n)\rfloor )$ where

$$\begin{aligned} f^n(x^n) = \sum _{t=1}^n f_t(x_t)\,, \end{aligned}$$

and $\delta (n,\epsilon )$ will be defined below.

We now have to show that this construction $g_n$ gives a correct lower-bound on the number of bits of randomness that can be extracted from $A^n$. Using the uniform continuity of *f*-weighted Rényi entropies (Lemma C.1), we find that for all $t \in [n]$ and all states of the form $\rho _{EXA} = {{\,\mathrm{\mathcal {M}}\,}}_t(\rho _{QE})$ with $\rho _{EQ} \in {{\,\mathrm{\mathcal {D}}\,}}_r^E({{\,\mathrm{\mathcal {H}}\,}}_{Q})$

$$\begin{aligned} H_\alpha ^{\uparrow ,f_t}(A|XE)_{\rho } \ge -(\alpha -1) (\log \eta _t)^2 \,, \end{aligned}$$

for $\alpha \in (1,1+1/\log \eta _t)$ where $\eta _t$ only depends on the dimension of *A* and on $\max _x f_t(x)$ and $\min _x f_t(x)$. Moreover, since $\eta _t$ takes only a finite set of values, we can bound $\eta _t \le \eta = \max _{t\in \mathbb {N}} \eta _t$.

Using Corollary 5.1, this implies that

$$\begin{aligned} H_\alpha ^{\uparrow , f_n}(A^n|X^nE)_{\rho } \ge - n (\alpha -1) (\log \eta )^2\,. \end{aligned}$$

for all state of the form $\rho _{EX^nA^n} = \mathcal {M}^n(\rho _{EQ^n})$ with $\rho _{EQ^n}\in {{\,\mathrm{\mathcal {D}}\,}}_r^E({{\,\mathrm{\mathcal {H}}\,}}_{Q^n})$. Finally, using [25, Theorem 1], this implies that the choice $\delta (n,\epsilon ) = n (\alpha -1) (\log \eta )^2 - \frac{\alpha }{\alpha -1}\log {1/\epsilon }$ leads to an $\epsilon$-secure protocol.

We now choose $\alpha = 1 + \tfrac{1}{\sqrt{n}}$ and $\epsilon _n = \frac{1}{n}$. Then, the average rate given by our construction is

$$\begin{aligned} \frac{1}{n} \mathbb {E}_{x^n \sim \otimes _{t=1}^n q_{X_t}^{\textrm{hon}}}[g(x^n)]&\ge \frac{1}{n} \sum _{t=1}^n \sum _{x} f_t(x)q^{\textrm{hon}}_{X_t}(x) - \frac{(\log \eta )^2}{\sqrt{n}} - \frac{2}{\sqrt{n}} \log {1/\epsilon _n} -O(1)\\&= \frac{1}{n} \sum _{t=1}^n h(\mathcal {M}_t,\mathcal {N}_t,\tau _t,q^{\textrm{hon}}_{X_t}) - O(\tfrac{\log n}{\sqrt{n}}) \end{aligned}$$

which yields the stated asymptotic limit. $\square$

**Application to the BB84 protocol**

For the BB84 protocol, it is known that in static cases the asymptotic key rate is given by the Shor-Preskill formula $1-2h(p)$ where *p* is error on the channel, $h(p) = -p \log _2 p - (1-p)\log _2 (1-p)$ is the binary entropy. Using a standard source replacement scheme, the protocol can be represented in its equivalent entanglement-based representation. In this case, the input space to a round of the protocol is the joint qubit space of Alice and Bob $Q = Q_AQ_B$ with $\mathcal {H}_{Q_A} = \mathcal {H}_{Q_B} = \mathbb {C}^{2}$.

Let us specify what $\mathcal {M},\mathcal {N},\tau$ are in this case. The map $\mathcal {M}$ corresponds to the following physical process: Alice and Bob each generate a uniformly random basis choice, measure in the corresponding *X* or *Z* basis and announce their basis publicly. Alice then randomly decides if the round is a test round, with some fixed probability $p_{\textrm{test}}$, and announces this publicly. In this case, Alice and Bob announce their measurement results publicly and Alice sets $A = \perp$; otherwise Alice assigns her measurement results to *A*. The variable *X* regroups all public announcements that were made.

Because of source replacement scheme, the state $\rho _{Q}$ that is input in a round of the protocol must satisfy $\hbox {tr}_{Q_B}[\rho _{Q}] = \frac{\mathbb {1}}{2}$. Therefore we have $Q' = Q_A$, $\mathcal {N} = \hbox {tr}_{Q_B}$ and $\tau = \frac{\mathbb {1}}{2}$.

We consider a family of honest implementations of the form $\rho ^{\textrm{hon}}_{Q} = (1-2p)\phi ^+ + \frac{p}{2} \mathbb {1}_Q$, with $\phi ^+$ the maximally entangled state and *p* a parameter that varies with time, to be defined later. This corresponds to an error in the *X* and *Z* basis of probability $\Pr [X_A\ne X_B] = \Pr [Z_A \ne Z_B] = p$. Let $q_{X}^{\textrm{hon}}$ be the corresponding distribution over public announcements. A standard result is that the asymptotic rate of randomness generation of the BB84 protocol is given by

$$\begin{aligned} h(\mathcal {M},\mathcal {N},\tau ,q_{X}^{\textrm{hon}}) = 1 - h(p) \end{aligned}$$

expressed in bits per key generation round.

Consider an honest distribution where *p* varies in times, so that $p=p_1=0.001$ for one third of the rounds and $p=p_2=0.1$ for the other two thirds, so that the average distribution corresponds to $\bar{p} = \frac{1}{3} p_1 + \frac{2}{3} p_2\approx 0.067$. Computing the corresponding time-adaptive and non-adaptive randomness generation rates and subtracting the cost of error correction given by $h(\bar{p})$, we find that the asymptotic secure key generation rates SKrate are

$$\begin{aligned} SKrate&= \frac{1}{3}(1- h(p_1)) + \frac{2}{3} (1-h(p_2)) - h(\bar{p}) \approx 0.329&\qquad \text {(time-adaptive)}\\ SKrate&= 1- 2 h(\bar{p}) \approx 0.291&\qquad \text {(not-adaptive)} \end{aligned}$$

This corresponds to an increase of 13% for time-adaptive methods compared to non adaptive methods.

## Data Availability

Data sharing is not applicable to this article as no datasets were generated or analyzed during the current study.

## A Some Properties of Completely Bounded Norms and a Proposition

In order to compute certain stabilized divergences involving a system *Q*, the system that acts as the ‘environment’ can often be assumed to have the same dimension as that of *Q*. Here we derive a general statement of this kind: in words, the optimization over environment systems *E* inherent in the cb-norm of some linear map is attained on ‘environments’ *E* that are isomorphic to the input system *A* of the map. Since for separable systems this statement is trivial we focus here on the finite dimensional one.

### Lemma A.1

Let $\Phi :\mathcal {B}(\mathcal {H}_A)\rightarrow \mathcal {X}$ be a linear map onto an arbitrary operator space $\mathcal {X}$, with $d_A:=\dim (\mathcal {H}_A)<\infty$. Then, for any $p\ge 1$,

$$\begin{aligned} \sup _{d\in \mathbb {N}}\Vert {{\,\textrm{id}\,}}_{d}\otimes \Phi \Vert _{\mathcal {S}_1(\mathbb {C}^d\otimes \mathcal {H}_A)\rightarrow \mathcal {S}_p[\mathbb {C}^d,\mathcal {X}]}=\Vert {{\,\textrm{id}\,}}_{d_A}\otimes \Phi \Vert _{\mathcal {S}_1(\mathbb {C}^{d_A}\otimes \mathcal {H}_A)\rightarrow \mathcal {S}_p[\mathbb {C}^{d_A},\mathcal {X}]} \end{aligned}$$

Note that when $d_A:=\dim (\mathcal {H}_A)=\infty$ the statement is clearly true in the sense that then the environment system *E* for which the supremum is achieved is isomorphic to system *A*. The proof of Lemma A.1 is a standard consequence of the Schmidt decomposition.

### Proof of Lemma A.1

For notational convenience, we denote the norm $\Vert .\Vert _{\mathcal {S}_p[\mathbb {C}^d,\mathcal {X}]}$ by $\Vert .\Vert _{\#}$ as well as the norm $\Vert .\Vert _{\mathcal {S}_1(\mathbb {C}^d\otimes \mathcal {H}_A)\rightarrow \mathcal {S}_p[\mathbb {C}^d,\mathcal {X}]}$ by $\Vert .\Vert _{1\rightarrow \#}$. Clearly $\Vert {{\,\textrm{id}\,}}_d\otimes \Phi \Vert _{1\rightarrow \#}$ is a non-decreasing sequence in *d*, hence it suffices to show that for $d\ge d_A$ it is non-increasing. Fix some $d\ge d_A$, then $\rho \mapsto \Vert ({{\,\textrm{id}\,}}_d\otimes \Phi )(\rho )\Vert _{\#}$ is convex, hence the maximum is achieved on the extremal operators in $\{\omega \in \mathcal {B}(\mathbb {C}^d\otimes \mathcal {H}_A)|\Vert \omega \Vert _1\le 1\}$, which are just rank-1 operators of the form $\omega =|\psi \rangle \langle \varphi |$:

$$\begin{aligned} \sup _{\Vert \omega \Vert _1\le 1} \Vert ({{\,\textrm{id}\,}}_d\otimes \Phi )(\omega )\Vert _{\#} = \sup _{|\varphi \rangle ,|\psi \rangle \in \mathbb {C}^d\otimes \mathcal {H}_A, \Vert |\varphi \rangle \Vert ,\,\Vert |\psi \rangle \Vert \le 1} \Vert ({{\,\textrm{id}\,}}_d\otimes \Phi )(|\psi \rangle \langle \varphi |)\Vert _{\#}\,. \end{aligned}$$

Now for any such $|\psi \rangle \in \mathbb {C}^d\otimes \mathcal {H}_A$, by Schmidt decomposition there exists a positive trace-normalized operator $\omega \in \mathcal {S}(\mathcal {H}_A)$ and a local isometry $U:\mathbb {C}^{d_A}\rightarrow \mathbb {C}^d$, s.t.

$$\begin{aligned} |\psi \rangle =(U\otimes \mathbb {1})\sum _{i=1}^{d_A}(\mathbb {1}\otimes \sqrt{\omega })(|i\rangle _{\mathbb {C}^{d_A}}\otimes |i\rangle _{\mathcal {H}_A}) \equiv (U\otimes \mathbb {1})|\sqrt{\omega }\rangle , \end{aligned}$$

i.e. $|\psi \rangle$ is a purification of some state $\omega$. Since $\#$ is invariant under local isometries (cf. Corollary 2.4), it follows that

$$\begin{aligned} \sup _{\psi ,\varphi } \Vert ({{\,\textrm{id}\,}}_d\otimes \Phi )(|\psi \rangle \langle \varphi |)\Vert _{\#}=\sup _{\omega ,\eta \in S(\mathcal {H}_A)}\Vert ({{\,\textrm{id}\,}}_{d_A}\otimes \Phi )(|\sqrt{\omega }\rangle \langle \sqrt{\eta }|)\Vert _\# \le \Vert {{\,\textrm{id}\,}}_{d_A}\otimes \Phi \Vert _{1\rightarrow \#}, \end{aligned}$$

since $$\Vert |\sqrt{\omega }\rangle \langle \sqrt{\eta }|\Vert _1\le \Vert \omega \Vert _1\Vert \eta \Vert _1\le 1$$. $$\square $$

Next, when considering the norm of a CP map, often one can restrict the optimization to run over over positive-semidefinite inputs, see. e.g. [26] for $q\rightarrow p$ and [10] for CB $q\rightarrow p$ norms. We generalize some of these statements here to norms of CP maps $\Phi :Q\rightarrow RS$ between operator-valued Schatten spaces.

### Lemma A.2

Let $\Phi :Q\rightarrow RS$ be a CP map. Then for $q\le r\le s$

$$\begin{aligned} \Vert \Phi \Vert _{cb, (Q:q)\rightarrow (R:r,S:s)}= \Vert \Phi \Vert ^+_{cb, (Q:q)\rightarrow (R:r,S:s)}, \end{aligned}$$

and moreover for $q \ge r,s$

$$\begin{aligned} \Vert \Phi \Vert _{cb,( Q:q)\rightarrow (R:r,S:s)}= \Vert \Phi \Vert ^+_{(Q:q)\rightarrow (R:r,S:s)}. \end{aligned}$$

These are generalizations of [10, Theorem 12 and 13 respectively], given our Theorem 3.4. Before giving their proof we require the following Lemma, which is a generalization of [10, Lemma 9].

### Proposition A.3

Let $r\le t\le s$ and $X\in B(\mathcal {H}_{123})$ be a contraction, then

$$\begin{aligned} \Vert C^*XD\Vert _{(r,t,s)}\le \Vert C^*C\Vert _{(r,t,s)}^{\frac{1}{2}}\Vert D^*D\Vert _{(r,t,s)}^{\frac{1}{2}}, \end{aligned}$$

where for notational simplicity we dropped the labeling in the notation, i.e. wrote $\Vert \cdot \Vert _{(r,t,s)}\equiv \Vert \cdot \Vert _{(1:r,2:t,3:s)}$, etc.

### Proof

By assumptions there exists, due to Lemma 3.1, $A,B\in S_{2x}(\mathcal {H}_1)$, with $\frac{1}{x}=\frac{1}{r}-\frac{1}{t}$ s.t. $\Vert A\Vert _{2x}=\Vert B\Vert _{2x}=1$, $A,B\ge 0$, and $Y,Z\ge 0$ such that

There further exist an isometries *V*, *W* s.t.

$$\begin{aligned} C=VY^{\frac{1}{2}}(A\otimes \mathbb {1}) \quad D=WZ^{\frac{1}{2}}(B\otimes \mathbb {1}), \end{aligned}$$

and hence $C^*XD=(A\otimes \mathbb {1})Y^{\frac{1}{2}}V^*XWZ^{\frac{1}{2}}(B\otimes \mathbb {1})$. So Lemma 3.1 together with [10][Lemma 9] yield

$$\begin{aligned} \Vert C^*XD\Vert _{(r,t,s)}\le \Vert Y^{\frac{1}{2}}V^*XWZ^{\frac{1}{2}}\Vert _{(t,t,s)}\le \Vert Y\Vert ^{\frac{1}{2}}_{(t,t,s)}\Vert Z\Vert ^{\frac{1}{2}}_{(t,t,s)} = \Vert C^*C\Vert ^{\frac{1}{2}}_{(r,t,s)}\Vert D^*D\Vert ^{\frac{1}{2}}_{(r,t,s)}, \end{aligned}$$

which is what we wanted to prove. $\square$

We may now give the proof of Lemma A.2.

### Proof

We first proof the first part. Fix some environment *E* and let $Q\in B(EQ)$. Let $Q=U|Q|^{\frac{1}{2}}|Q|^{\frac{1}{2}}$ be its polar decomposition. Then

$$\begin{aligned} 0 < \begin{pmatrix} U|Q|^{\frac{1}{2}} \\ |Q|^{\frac{1}{2}} \end{pmatrix} (|Q|^{\frac{1}{2}}U^*&\quad |Q|^{\frac{1}{2}}) = \begin{pmatrix} U|Q|U^* & \quad Q \\ Q^* & \quad |Q| \end{pmatrix}. \end{aligned}$$

Now since $\Phi$ is CP, ${{\,\textrm{id}\,}}_2\otimes {{\,\textrm{id}\,}}_E\otimes \Phi$ is positive and hence so is

$$\begin{aligned} \begin{pmatrix} ({{\,\textrm{id}\,}}_E\otimes \Phi )(U|Q|U^*) & \quad ({{\,\textrm{id}\,}}_E\otimes \Phi )(Q) \\ ({{\,\textrm{id}\,}}_E\otimes \Phi )(Q^*) & \quad ({{\,\textrm{id}\,}}_E\otimes \Phi )(|Q|) \end{pmatrix} >0. \end{aligned}$$

Now it is well known [14] that this block matrix being positive is equivalent to

17 $$\begin{aligned} ({{\,\textrm{id}\,}}_E\otimes \Phi )(Q) = ({{\,\textrm{id}\,}}_E\otimes \Phi )(U|Q|U^*)^{\frac{1}{2}}X(({{\,\textrm{id}\,}}_E\otimes \Phi )(|Q|))^{\frac{1}{2}} \end{aligned}$$

for some contraction *X*. Now directly (17) and Proposition A.3 yield the desired result, since $q\le r\le s$ and

$$\begin{aligned} \Vert ({{\,\textrm{id}\,}}_E\otimes \Phi )(Q) \Vert _{(E:q,R:r,S:s)}&= \Vert ({{\,\textrm{id}\,}}_E\otimes \Phi )(U|Q|U^*)^{\frac{1}{2}}X(({{\,\textrm{id}\,}}_E\otimes \Phi )(|Q|))^{\frac{1}{2}}\Vert _{(E:q,R:r,S:s)} \\  &\le \Vert ({{\,\textrm{id}\,}}_E\otimes \Phi )(U|Q|U^*)\Vert ^{\frac{1}{2}}_{(E:q,R:r,S:s)}\Vert ({{\,\textrm{id}\,}}_E\otimes \Phi )(|Q|)\Vert ^{\frac{1}{2}}_{(E:q,R:r,S:s)} \\&\le \Vert {{\,\textrm{id}\,}}_E\otimes \Phi \Vert ^+_{(E:q,Q:q)\rightarrow (E:q,R:r,S:s)} \Vert U|Q|U^*\Vert ^{\frac{1}{2}}_{(E:q,Q:q)}\Vert |Q|\Vert ^{\frac{1}{2}}_{(E:q,Q:q)} \\  &= \Vert {{\,\textrm{id}\,}}_E\otimes \Phi \Vert ^+_{(E:q,Q:q)\rightarrow (E:q,R:r,S:s)}\Vert Q\Vert _{q}, \end{aligned}$$

where in the last equality we used unitary invariance in of the Schatten-$p-$norm. Taking the supremum over *E* gives the result.

To prove the second part we use the fact that the SWAP operator is a complete contraction, see (7), hence since $q\ge r,s$

$$\begin{aligned} \Vert \Phi \Vert _{cb,Q:q\rightarrow (R:r,S:s)}&= \sup _E\sup _{X_{EQ}}\frac{\Vert ({{\,\textrm{id}\,}}_E\otimes \Phi )(X_{EQ})\Vert _{(E:q,R:r,S:s)}}{\Vert X_{EQ}\Vert _{(E:q,Q:q)}} \\  &= \sup _E\sup _{X_{EQ}}\frac{\Vert ({{\,\textrm{id}\,}}_E\otimes \Phi )(X_{EQ})\Vert _{(E:q,R:r,S:s)}}{\Vert X_{QE}\Vert _{q}} \\  &\le \sup _E\sup _{X_{QE}}\frac{\Vert (\Phi \otimes {{\,\textrm{id}\,}}_E)(X_{QE})\Vert _{(R:r,S:s,E:q)}}{\Vert X_{QE}\Vert _{q}} \\  &\le \Vert \Phi \Vert ^+_{Q:q\rightarrow (R:r,S:s)}, \end{aligned}$$

where the first inequality are two applications of the SWAP operator and the last inequality is Theorem 4.1. $\square$

## B Additivity on Reduced State Space

Now we are in a position to prove Theorem 4.16 starting from Theorem 4.11 using an argument from [25]. There it was used to prove a version of Corollary 5.1 under *n*-fold tensor products of channels.

### Proof of Theorem 4.16

To fix *n* linear constraints fix some CPTP channels $\{\mathcal {N}_i:Q_i\rightarrow Q^\prime _i\}_{i=1}^n$ and states $\{\tau _i\in \mathcal {D}({{\,\mathrm{\mathcal {H}}\,}}_{Q^\prime _i})\}_{i=1}^n$. Recall the definitions of $Q^n:=Q_1...Q_n$, $\mathcal {D}_r({{\,\mathrm{\mathcal {H}}\,}}_{Q})$ and $\mathcal {D}^E_r({{\,\mathrm{\mathcal {H}}\,}}_{Q})$. WLOG assume that none of the $\Phi _i\ne 0,$ else the statement trivially holds.

To do this we first recall, that due to Lemma A.1 and its proof it holds that

$$\begin{aligned} \Vert \Phi \Vert _{r,cb,Q:1\rightarrow (R:1,S:p)}&= \sup _{E}\sup _{\rho _{EQ}\in \mathcal {D}^E_r({{\,\mathrm{\mathcal {H}}\,}}_Q)}\Vert ({{\,\textrm{id}\,}}_E\otimes \Phi )(\rho _{EQ})\Vert _{(E:1,R:1,S:p)} \\  &= \sup _{\rho \in \mathcal {D}_r({{\,\mathrm{\mathcal {H}}\,}}_Q)}\Vert ({{\,\textrm{id}\,}}_{\tilde{Q}}\otimes \Phi )(|\sqrt{\rho }\rangle \langle \sqrt{\rho }|_{\tilde{Q}Q})\Vert _{(\tilde{Q}:1,R:1,S:p)} \\&=:\sup _{\rho _\in \mathcal {D}_r({{\,\mathrm{\mathcal {H}}\,}}_{Q})}g_p^{\Phi }(\rho _Q) \\&=\sup \{g_p^\Phi (\rho _Q)| \rho _Q\ge 0, \mathcal {N}(\rho _Q)=\tau \}, \end{aligned}$$

where $\tilde{Q}$ is isomorphic to *Q* and $|\sqrt{\rho }\rangle _{\tilde{Q}Q}=(\mathbb {1}_{\tilde{Q}}\otimes \sqrt{\rho }_Q)\sum _{i}|ii\rangle _{\tilde{Q} Q}$ is a (canonical) purification of $\rho _Q\in \mathcal {D}_r({{\,\mathrm{\mathcal {H}}\,}}_{Q})$ into $\tilde{Q} Q$. This is because the extremal points of the linear constrained $\mathcal {D}_r^E({{\,\mathrm{\mathcal {H}}\,}}_{Q}):=\{\rho \in \mathcal {D}({{\,\mathrm{\mathcal {H}}\,}}_{EQ})|(\mathcal {N}\circ \hbox {tr}_E)(\rho _{EQ})=\tau \}$ are nothing but the set of purifications of all $\rho \in \mathcal {D}_r({{\,\mathrm{\mathcal {H}}\,}}_{Q})$, by construction. To get the last line we used that the linear constraint already enforces $\hbox {Tr}[\rho ]=\hbox {Tr}[\mathcal {N}(\rho )]=\hbox {Tr}[\tau ]=1$, since $\mathcal {N}$ is TP.

Hence the statement of the theorem is equivalent to

18 $$\begin{aligned} \sup _{\rho \in \mathcal {D}_r({{\,\mathrm{\mathcal {H}}\,}}_{Q^n})}g_p^{\otimes _{i=1}^n\Phi _i}(\rho ) = \prod _{i=1}^n \sup _{\rho _i\in \mathcal {D}_r({{\,\mathrm{\mathcal {H}}\,}}_{Q_i})} g^{\Phi _i}_p(\rho _i). \end{aligned}$$

Since for operators $\rho _i\in \mathcal {D}_r({{\,\mathrm{\mathcal {H}}\,}}_{Q_i})$ it clearly holds that $\otimes _{i=1}^n\rho _i\in \mathcal {D}_r({{\,\mathrm{\mathcal {H}}\,}}_{Q^n})$ it actually suffices to prove that the LHS in (18) is upper bounded by the RHS. To do so the authors in [25] have shown that in the finite dimensional setting, the above convex optimization problem is equal to its dual, i.e.

19 $$\begin{aligned}&\sup _{\rho \in {{\,\textrm{Pos}\,}}(\mathcal {H}_Q)}\{g^\Phi _p(\rho )|\mathcal {N}(\rho )=\tau \} \nonumber \\&\quad = \inf _{\Sigma \in {{\,\textrm{Pos}\,}}(\mathcal {H}_{Q^\prime })}\{\hbox {Tr}[\Sigma \tau ]| g^\Phi _p(\rho )\le \hbox {Tr}[\Sigma \mathcal {N}(\rho )] \forall \rho \in {{\,\textrm{Pos}\,}}(\mathcal {H}_Q)\}. \end{aligned}$$

This follows by showing that there exists a dual feasible $\Sigma \in {{\,\textrm{Pos}\,}}(\mathcal {H}_{Q^\prime })$, i.e. one that satisfies

$$\begin{aligned} g_p^\Phi (\rho )<\hbox {Tr}[\Sigma \mathcal {N}(\rho )] \ \forall \rho \in {{\,\textrm{Pos}\,}}(\mathcal {H}_Q). \end{aligned}$$

For the convenience of the reader we will give a simple proof. Note first that both sides are positive homogeneous in $\rho$, hence it suffices to show it for all $\rho \in \mathcal {D}({{\,\mathrm{\mathcal {H}}\,}}_{Q})$. Let $\Sigma =C\mathbb {1}_{Q^\prime }$, then the RHS becomes $\hbox {Tr}[\Sigma \mathcal {N}(\rho )]=C$, since $\mathcal {N}$ is CPTP and this inequality holds strictly, by definition for $C =\Vert \Phi \Vert _{cb,Q:1\rightarrow (R:1,S:p)} + 1<\infty$ by assumption. Now that we have strong duality, let $\Sigma _i$ be a feasible point of the dual problem, i.e. for any $0\ne \rho \in {{\,\textrm{Pos}\,}}(\mathcal {H}_{Q_i})$, $0<g_p^{\Phi _i}(\rho )\le \hbox {Tr}[\mathcal {N}^*_i(\Sigma _i)\rho ]$, since $\Phi _i\ne 0$. It follows that $\mathcal {N}^*(\Sigma _i) > 0$. Define the hence strictly positive $V_i:=\sqrt{\mathcal {N}^*_i(\Sigma _i)}$ and with it the CP map $\Phi _i^\prime (\cdot ):=\Phi _i(V_i^{-1}\cdot V_i^{-1})$. Now we see that

$$\begin{aligned} g_p^{\Phi _i}(\rho )&\le \hbox {Tr}[\mathcal {N}^*_i(\Sigma )\rho ] \quad&\forall \rho \in {{\,\textrm{Pos}\,}}(\mathcal {H}_{Q_i}), \\ \Leftrightarrow g_p^{\Phi ^\prime _i}(\rho ^\prime )&\le \hbox {Tr}[\rho _i^\prime ] \quad&\forall \rho ^\prime \in {{\,\textrm{Pos}\,}}(\mathcal {H}_{Q_i}), \\ \Leftrightarrow g_p^{\Phi ^\prime _i}(\rho ^\prime )&\le 1&\forall \rho ^\prime \in \mathcal {D}({{\,\mathrm{\mathcal {H}}\,}}_{Q_i}), \\ {\Rightarrow } g_p^{\otimes _{i=1}^n\Phi ^\prime _i}(\rho _n^\prime )&\le 1 \quad&\forall \rho _n^\prime \in \mathcal {D}({{\,\mathrm{\mathcal {H}}\,}}_{Q^n}), \\ \Leftrightarrow g_p^{\otimes _{i=1}^n\Phi ^\prime _i}(\rho _n^\prime )&\le \hbox {Tr}[\rho _n^\prime ] \quad&\forall \rho _n^\prime \in {{\,\textrm{Pos}\,}}(\mathcal {H}_{Q^n}), \\ \Leftrightarrow g_p^{\otimes _{i=1}^n\Phi _i}(\rho _n)&\le \hbox {Tr}[\otimes ^n_{i=1}\mathcal {N}^*_i(\Sigma _i)\rho _n] = \hbox {Tr}[(\otimes _{i=1}^n\Sigma _i)(\otimes _{i=1}^n\mathcal {N}_i)(\rho _n)] \quad&\forall \rho _n\in {{\,\textrm{Pos}\,}}(\mathcal {H}_{Q^n}). \end{aligned}$$

where $\rho ^\prime :=V_i\rho V_i$ and $\rho _n=(\bigotimes _{i=1}^nV_i^{-1})\rho ^\prime _n(\bigotimes _{i=1}^nV_i^{-1})$ , where the implication above followed from Theorem 4.11. So we have shown that $\otimes _{i=1}^n \Sigma _i$ is dual feasible. In addition, the objective value for $\otimes _{i=1}^n \Sigma _i$ is $\prod _{i=1}^n \hbox {Tr}[\Sigma _i \tau _i]$. Taking the infimum over all feasible $\Sigma _i$ for $i\in [n]$ yields the claim due to strong duality (19). $\square$

## C Uniform Continuity for *f*-weighted Renyi Entropies

### Lemma C.1

Consider systems *AE* with finite-dimensional Hilbert spaces, a classical system *X* with basis elements labeled by $\mathbb {X}$ and a function $f:\mathbb {X}\rightarrow \mathbb {R}$. Define $\eta _0 = |A| (2^{\max _x f(x)} + 2^{-\min _x f(x)}) + 1$. For $\alpha \in (1,1+\frac{1}{\log \eta _0})$, we have for all states $\rho \in {{\,\mathrm{\mathcal {D}}\,}}({{\,\mathrm{\mathcal {H}}\,}}_{EXA})$

$$\begin{aligned} H(A|XE)_{\rho }&- \mathbb {E}_{x \sim \rho _X}[f(X)] - (\alpha -1) (\log \eta _0)^2 \le H^{\uparrow ,f}_\alpha (A|XE)_{\rho } \le H(A|XE)_{\rho } \\&- \mathbb {E}_{x \sim \rho _X}[f(X)]\,. \end{aligned}$$

### Proof

We can get this result from the uniform continuity of Rényi divergences which are defined for $\alpha \in (0,1)\cup (1,\infty )$ as

$$\begin{aligned}&D_\alpha (\rho \Vert \sigma ) = \frac{\alpha }{1-\alpha } \log \Vert \sigma ^{\tfrac{1-\alpha }{2\alpha }} \rho \sigma ^{\tfrac{1-\alpha }{2\alpha }}\Vert _{\alpha }\\&D'_\alpha (\rho \Vert \sigma ) = \frac{1}{\alpha -1} \log \hbox {Tr}[\rho ^{\alpha } \sigma ^{1-\alpha }] \end{aligned}$$

and for $\alpha =0$ as $D'_0(\rho \Vert \sigma ) = \lim _{\alpha \rightarrow 0}D'_\alpha (\rho \Vert \sigma )$. We note that

$$\begin{aligned} H^{\uparrow ,f}_\alpha (A|XE)_{\rho } = - \min _{\sigma \in {{\,\mathrm{\mathcal {D}}\,}}({{\,\mathrm{\mathcal {H}}\,}}_{XE})} D_\alpha (\rho _{AXE} \Vert 2^{-f_X}\cdot \sigma _{XE}) \ge - D_\alpha (\rho _{AXE} \Vert 2^{-f_X}\cdot \rho _{XE}) \end{aligned}$$

and

$$H(A|XE)_{\rho } - \mathbb {E}[f(X)] = - D(\rho _{AXE}\Vert 2^{-f_X}\cdot \rho _{XE}) = - \min _{\sigma \in {{\,\mathrm{\mathcal {D}}\,}}({{\,\mathrm{\mathcal {H}}\,}}_{XE})} D_{\alpha }(\rho _{AXE}\Vert 2^{-f_X}\cdot \rho _{XE}).$$

In [11, Lemma B.8 and Eq. (82)] it is proven that, for all $\alpha \in (1,1+\log (\eta ))$,

$$\begin{aligned} D(\rho _{AXE} \Vert 2^{-f_X}\rho _{XE}) \le D_\alpha (\rho _{AXE} \Vert 2^{-f_X}\rho _{XE}) \le D(\rho _{AXE} \Vert 2^{-f_X}\rho _{XE}) + (\alpha -1) (\log \eta )^2 \end{aligned}$$

with $$\eta = 2^{D'_2(\rho _{AXE} \Vert 2^{-f_X} \rho _{XE})} + 2^{-D_0(\rho _{AXE} \Vert 2^{-f_X} \rho _{XE})} + 1$$.

This yields the desired continuity statement, provided we can bound $\eta$ in a state independent way. For this, observe that for all $\alpha \in (1,\infty )$,

$$\begin{aligned} D'_\alpha (\rho _{AXE}\Vert 2^{-f_{X}}\rho _{XE})&= \frac{1}{\alpha -1} \log \hbox {Tr}[\rho _{AXE}^{\alpha } \rho _{XE}^{1-\alpha } 2^{(\alpha -1)f_{X}} ]\\&\le \frac{1}{\alpha -1} \log ( \hbox {Tr}[\rho _{AXE}^{\alpha } \rho _{XE}^{1-\alpha } ] 2^{(\alpha - 1) \max _{x} f(x)}) \\&\le \log |A| + \max _x f(x)\,. \end{aligned}$$

Similarly, $-D'_\alpha (\rho _{AXE} \Vert 2^{-f_X}\rho _{XE}) \le \log \hbox {tr}[2^{-f_X} \rho _{XE}] \le \log |A| - \min _x f(x)$. This implies that

$$\begin{aligned} \log \eta \le \log (|A|2^{\max _x |f(x)|} + |A|2^{-\min _x f(x)}+1). \\ \end{aligned}$$

$$\square $$

## Footnotes

1 The inequality proved in [17] holds in fact more generally for channels satisfying a certain non-signalling property.
